# A biomineral-inspired approach of synthesizing colloidal persistent phosphors as a multicolor, intravital light source

**DOI:** 10.1126/sciadv.abo6743

**Published:** 2022-07-29

**Authors:** Fan Yang, Xiang Wu, Han Cui, Zihao Ou, Shan Jiang, Sa Cai, Qi Zhou, Bryce G. Wong, Hans Huang, Guosong Hong

**Affiliations:** ^1^ Department of Materials Science and Engineering, Stanford University, Stanford, CA 94305, USA.; ^2^ Wu Tsai Neurosciences Institute, Stanford University, Stanford, CA 94305, USA.

## Abstract

Many in vivo biological techniques, such as fluorescence imaging, photodynamic therapy, and optogenetics, require light delivery into biological tissues. The limited tissue penetration of visible light discourages the use of external light sources and calls for the development of light sources that can be delivered in vivo. A promising material for internal light delivery is persistent phosphors; however, there is a scarcity of materials with strong persistent luminescence of visible light in a stable colloid to facilitate systemic delivery in vivo. Here, we used a bioinspired demineralization (BID) strategy to synthesize stable colloidal solutions of solid-state phosphors in the range of 470 to 650 nm and diameters down to 20 nm. The exceptional brightness of BID-produced colloids enables their utility as multicolor luminescent tags in vivo with favorable biocompatibility. Because of their stable dispersion in water, BID-produced nanophosphors can be delivered systemically, acting as an intravascular colloidal light source to internally excite genetically encoded fluorescent reporters within the mouse brain.

## Teaser

A bioinspired approach produces bright afterglow colloids that can excite endogenous fluorescent proteins for brain imaging.

## INTRODUCTION

Light is used in a wide range of methods in biology and medicine, such as fluorescence imaging (*1*), optogenetics (*2*), photoactivatable gene editing (*3*), light-controlled immunotherapy (*4*), and photochemotherapy for cancers and viral infections (*5*). A critical challenge of applying light in vivo, such as deep-brain imaging and optogenetics, arises from the poor penetration of photons in biological tissue due to scattering and absorption (*1*). Therefore, delivering photons deep into the body from an external light source requires invasive procedures, such as craniotomy, to surgically remove overlying tissues.

Besides conventionally used external light sources, internally delivered light sources represent an arising opportunity to mitigate the challenges associated with the poor tissue penetration of photons. On the one hand, nanofabricated light sources in a flexible and stretchable platform provide a minimally invasive strategy for delivering an internal light source in vivo (*6*, *7*). On the other hand, microparticles and nanoparticles with persistent luminescence, also known as the afterglow, represent another potential approach for internal light delivery owing to their ability to store photon energy in their chemical structures. Although afterglow imaging with these materials has attracted substantial research interest (*8*–*10*), conventional afterglow materials emit red to near-infrared (NIR) light to facilitate imaging with deep tissue penetration (*8*, *9*, *11*). In contrast, materials with a strong afterglow in the short-wavelength visible spectrum are needed to fulfill their roles as a light source to excite fluorescent proteins, opsins, photoswitchable Cas9 (psCas9), etc., due to the activation spectrum of these proteins in the visible range (*2*, *12*, *13*). In addition, these short-wavelength light sources must have good colloidal stability in physiological aqueous environments to facilitate their delivery in vivo, preferably via the circulatory system. Despite recent efforts in developing short-wavelength afterglow materials for biological applications (*14*, *15*), the decay lifetimes and brightness of these materials remain to be improved. Thus, there is still a scarcity of afterglow nanomaterials with long lifetimes, sufficiently short wavelengths (especially blue), and colloidal stability to enable in vivo use as an internal light source.

We argue that the lack of short-wavelength afterglow colloids reflects an intrinsic gap between wet-chemical and solid-state reactions for synthesizing persistent luminescence phosphors. On the one hand, wet-chemical approaches (e.g., the sol-gel method) have enabled the synthesis of chromium-doped gallate (ZnGa_2_O_4_:Cr^3+^, 695 nm), semiconducting polymers (780 nm), and rare-earth doped nanoparticles (NaLnF_4_:RE, >1000 nm) with sufficient brightness and biocompatibility for deep-tissue afterglow imaging (*8*–*10*, *16*–*19*). However, the long wavelengths of these materials are energetically unfavorable to excite fluorescent proteins, opsins, and psCas9. It is hypothesized that point defects necessary for strong persistent luminescence in the short-wavelength visible spectrum are thermodynamically and kinetically unfavorable to form at low processing temperatures, thus making conventional sol-gel methods incapable of synthesizing blue-shifted afterglow nanoparticles (*20*). On the other hand, solid-state reactions enable the synthesis of inorganic phosphors with tunable wavelengths down to 400 nm and strong persistent luminescence by facilitating uniform doping of color centers in host materials of desirable polymorphs (*21*). However, solid-state–synthesized phosphors remain refractory to wet-chemical methods; moreover, their large sizes (>10 μm) prohibit their use in a living body (*20*). Although mechanical milling represents a plausible route to produce nanoparticles from solid-state products, the plastic deformation process introduces undesired built-in stresses and dislocations into the resulting nanoparticles (*22*). These built-in stresses and dislocations lead to mechanical quenching and defect-induced quenching of milled phosphor nanoparticles (*23*, *24*), thus making mechanical milling an unfavorable approach for producing colloidal light sources from solid-state precursor particles.

Here, we report a bioinspired demineralization (BID) approach to synthesize stable colloidal solutions of solid-state phosphors with tunable wavelengths and remarkable afterglow intensity. The BID approach is a generalizable wet-chemical method to produce colloidal nanophosphors from high-temperature, solid-state reactions, thus bridging the gap between these two conventional, yet mutually incompatible strategies (i.e., sol-gel versus solid-state) discussed above. In addition, the BID approach is a much milder alternative to mechanical milling, thereby avoiding the built-in stress and dislocations induced during milling that would otherwise quench the afterglow. Specifically, the BID approach is inspired by the strategy of biomineralization in nature: Biominerals, such as apatite in the dental enamel, can be gradually dissolved to nanostructures in a mildly acidic environment yet are resistant to further dissolution (*25*). In this work, we demonstrate that the kinetic stability of nanostructures is also prevalent in solid-state phosphors and apply this universal strategy to a wide array of phosphors. Specifically, the BID approach can produce stable colloidal solutions of silicates, aluminates, and sulfides with diameters down to 20 nm and afterglow wavelengths from 470 to 650 nm. These nanophosphor colloids preserve the high crystallinity, bright luminescence, long afterglow, and specific wavelength of their micrometer-sized precursors from solid-state reactions. We demonstrate these water-soluble materials as colloidal light sources that can be delivered via intravenous injection, enabling internal excitation of genetically encoded fluorescent proteins in vivo with advanced tissue penetration.

## RESULTS

### A BID approach of producing colloidal solutions of nanophosphors

**Fig. 1.**
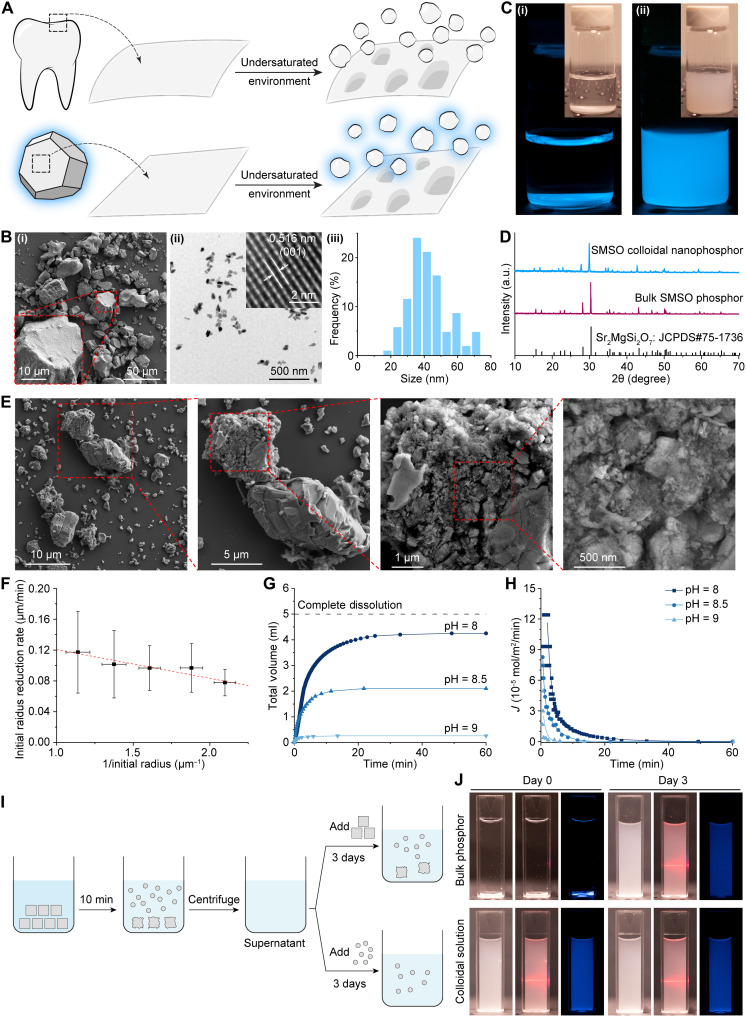
BID approach for synthesizing colloidal SMSO solutions. (**A**) Schematics showing the BID mechanism for dissolving tooth enamel (top) and sparingly soluble phosphors (bottom) into nanoparticles in an undersaturated solution. (**B**) SEM (i) and transmission electron microscope (TEM) (ii) images of the bulk SMSO phosphor and its colloidal nanoparticles, respectively. The inset in (ii) shows the high-resolution TEM image of an SMSO nanoparticle. A histogram showing the size distribution of SMSO colloids is shown in (iii). (**C**) Afterglow images and bright-field images (insets) of an aqueous suspension of bulk SMSO phosphor (i) and its stable colloidal solution of nanophosphors (ii). (**D**) X-ray diffraction (XRD) spectra of bulk SMSO phosphor and its colloidal nanoparticles. An average domain size of 52 nm was obtained by analyzing peak widths in the XRD spectrum of SMSO colloids with the Scherrer equation. a.u., arbitrary units. (**E**) SEM images of bulk SMSO particles after reaching a metastable equilibrium. (**F**) Plot showing the average instantaneous dissolution rate of SMSO particles as a function of their average inverse radius. The data are represented as mean values ± standard deviation (SD). (**G**) Plots of the titrant volume as a function of time for kinetically preserved dissolution of SMSO at different pH. (**H**) Plots of the flux rate (*J*) as a function of time for SMSO dissolution at different undersaturations. (**I**) Schematic showing the steps of an experimental procedure that verifies the BID mechanism. (**J**) Bright-field image (left), Tyndall effect (middle), and afterglow image (right) of colloidal solutions prepared under different conditions (see Materials and Methods).

Satisfying the requirement for bright luminescence and tunable wavelengths prohibits the use of sol-gel methods and necessitates solid-state reactions at elevated temperatures to produce persistent phosphors with desired point defects in host materials of specific phases (*20*). However, solid-state phosphors prepared at high temperatures are usually >10-μm particles composed of water-insoluble materials. As discussed in Introduction, these solid-state phosphors are refractory to ball milling due to mechanical and dislocation-induced quenching effects. To mitigate this challenge, we leveraged a unique phenomenon found in nature in the process of demineralization. Demineralization of naturally occurring biominerals (e.g., tooth enamel and seashells) features a self-preservation behavior, which exhibits the kinetic stability of biomineral nanoparticles in an aqueous solution despite their thermodynamic metastable nature (*25*, *26*). We hypothesize that this kinetic stability of nanostructures is also applicable to solid-state phosphors with a low water solubility, such as sparingly soluble silicates and aluminates (Fig. 1A) (*27*, *28*). This kinetic stability, despite thermodynamic metastability, is the basis of the BID technique.

Specifically, the BID technique contrasts conventional wet-chemical etching, which usually exhibits self-accelerating dissolution (such as the dissolution of a cane sugar crystal in water). Smaller particles have higher specific surface energy and thus greater solubility than larger ones, which is well known as Ostwald ripening (*29*). This behavior prohibits the use of conventional wet-chemical etching to produce a stable colloidal solution of persistent phosphors, especially those <50 nm, without complete dissolution of the colloids. In contrast, biominerals, which share similar chemical compositions and sparing solubility as solid-state phosphors, exhibit suppressed dissolution of their nanosized crystallites in undersaturated solutions (*25*, *26*). Specifically, the dissolution of biominerals is facilitated by the formation and growth of pits on the surfaces. It has been theoretically predicted and experimentally validated that the rate of dissolution, *R*, is related to the radius of the pit, *r*, as follows (*27*)

$$R=R_{\infty }(1-\frac{r^{*}}{r})$$ (1)

where *R*_∞_ is the dissolution rate at *r* → ∞, which is usually achieved in bulk material. r* is the critical radius of the pit, which determines the size of kinetically preserved nanoparticles and is related to the surface energy and Gibbs free energy of dissolution as follows

$$r^{*}=-\frac{\gamma_{\text{SL}}\Omega }{\Delta g}$$ (2)

Here, γ_SL_ represents the interfacial tension of a solid in the solution, Ω is the surface area of each dissolution unit, and Δ*g* is the Gibbs free energy of dissolution per dissolution unit. Sparingly soluble solids such as biominerals and inorganic phosphors always have much larger interfacial tension than soluble salts (*30*), thus resulting in large values of r* in the range of 10 to 100 nm. Therefore, we hypothesize that this BID approach can provide a generalizable method to produce nanophosphors with targeted size distributions determined by r* (Fig. 1A).

In the BID process, we used a citrate buffer to mimic the undersaturated environment that etches biominerals to produce colloid solutions of solid-state nanophosphors (see Materials and Methods). Citrate buffer at pH < 10 provides a negative Δ*g* to satisfy Eq. 2 (figs. S1 to S5) instead of serving the role of a surfactant in conventional mechanical milling methods. We selected strontium magnesium silicate doped with Eu^2+^ and Dy^3+^ (Sr_2_MgSi_2_O_7_:Eu,Dy, SMSO) as a representative example of persistent phosphors, owing to its strong and long-lasting blue afterglow that provides excitation for many light-activated proteins such as the yellow fluorescent protein (YFP), stable step-function opsins (SSFO), and psCas9 (*3*, *12*, *13*, *31*). SMSO with a strong afterglow cannot be synthesized via a sol-gel method due to crystallinity and doping requirements, thus generally resulting in micrometer-sized particles that cannot form a stable colloidal suspension in water (*32*).

In our experiments, SMSO synthesized in solid-state reactions began with an average size of >10 μm, as evidenced by scanning electron microscope (SEM) images (Fig. 1Bi). The large size distribution of these SMSO microparticles prohibited a stable suspension in water, as evidenced by a blue afterglow from the precipitates instead of the supernatant (Fig. 1Ci). In contrast, the BID approach produced a stable colloidal solution of SMSO nanophosphors with a strong and uniform blue afterglow (Fig. 1Cii) and a size distribution of 43 ± 11 nm (Fig. 1, B, ii and iii, and D). We used Fourier transform infrared (FTIR) spectroscopy, nuclear magnetic resonance (NMR) spectroscopy, and ultraviolet (UV)–visible absorption spectroscopy to fully characterize the products of the BID process (figs. S1 to S5).

### Mechanistic study of the BID technique for producing colloidal solutions of nanophosphors

We next sought to validate the mechanism of kinetic preservation to produce SMSO colloids. First, surface roughening represents a notable hallmark of the self-preserved demineralization process of biominerals (*25*). We compared the SEM images of large SMSO particles before and after the BID process (Fig. 1, Bi and E). In contrast to the smooth surface before, SMSO particles after the BID process exhibited much rougher surfaces, featuring nanostructures such as particles and troughs with sizes ≤100 nm, similar to those found during natural demineralization. During surface roughening, every single micrometer-sized SMSO particle shed ~10^6^ nanoparticles into the solution (see Supplementary Text).

Second, according to Eq. 1, the BID model predicts a size-dependent dissolution rate, approaching zero for colloidal nanoparticles with sizes close to or smaller than r*. To validate this dependence, we performed real-time confocal fluorescence microscopy to monitor the dissolution rate during the BID process (see Materials and Methods). A clear size-dependent dissolution rate was observed, with a decreasing size-reduction rate for smaller colloidal nanoparticles (Fig. 1F). To quantify the size-dependent dissolution rate, we applied a previously reported constant-composition (CC) technique (*25*) to extract the dissolution flux rate against time at different undersaturations determined by the pH of the solution (see Materials and Methods; Fig. 1, G and H, and figs. S6 and S7). The CC dissolution curves reached a plateau before complete dissolution, thus indicating the creation of metastable states for both undissolved large SMSO particles with roughened surfaces and colloidal nanoparticles shed into the solution.

Third, we hypothesized that, in the same undersaturated solution, only nanoparticles with sizes ≤ r* can be kinetically preserved, while bulk particles can still be dissolved (Fig. 1I). We experimentally verified that fresh, bulk SMSO particles were etched to nanoparticles in the same undersaturated solution despite a suppressed reaction for previously immersed particles (Fig. 1J, top). Furthermore, we found that SMSO colloidal nanoparticles produced in an undersaturated suspension resisted further dissolution after separation and reimmersion in a solution of the same undersaturation (Fig. 1J, bottom, and fig. S8).

Last, we also verified minimal dissolution of bulk SMSO particles in pure water without citrate (pH 7), thus confirming the importance of rationally designed undersaturated environments in the BID method (fig. S9). In summary, we have validated the kinetic preservation mechanism to produce stable colloidal solutions of SMSO nanophosphors via the BID approach.

### BID-produced SMSO colloids exhibit strong emission with a long luminescence lifetime

**Fig. 2.**
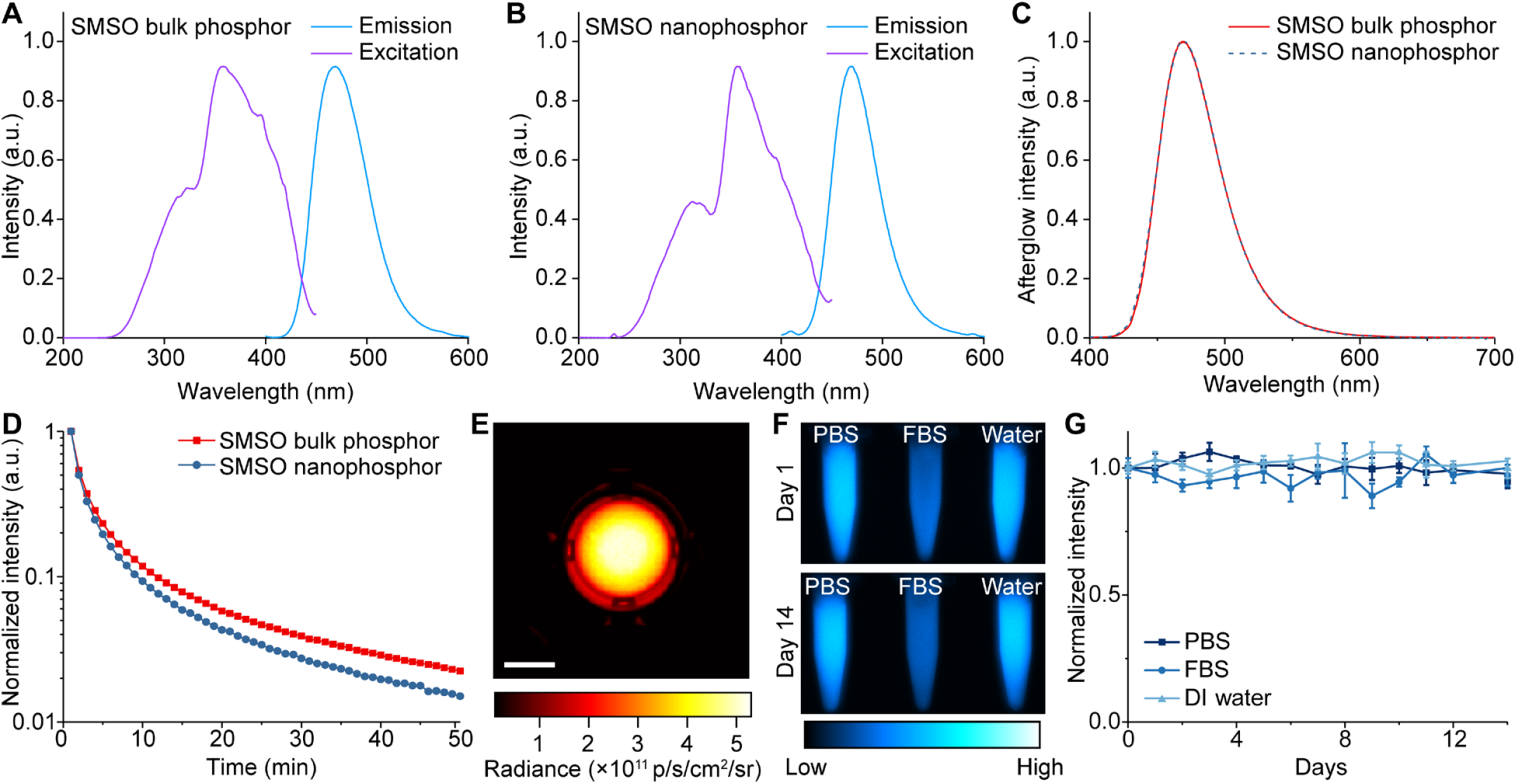
Strong and persistent afterglow of BID-produced SMSO nanophosphor colloids. (**A**) Excitation and emission spectra of untreated, bulk SMSO phosphor. (**B**) Excitation and emission spectra of a colloidal solution of SMSO nanophosphors. (**C**) Afterglow spectra of SMSO bulk phosphor and colloidal nanophosphor. (**D**) Afterglow curves of SMSO bulk phosphor and colloidal nanophosphor. (**E**) Afterglow image of a colloidal solution of SMSO nanophosphors (493 nM) in a 48-well plate acquired by the IVIS imaging system. Scale bar, 0.5 cm. (**F**) Afterglow images of colloidal solutions of SMSO nanophosphors in phosphate-buffered saline (PBS) (left), fetal bovine serum (FBS) (middle), and water (right) at day 1 (top) and day 14 (bottom). The decrease in afterglow intensity for FBS is due to the absorption of FBS at the emission wavelengths of SMSO colloids and does not reflect the instability of SMSO afterglow (fig. S10). (**G**) Chronic stability of normalized afterglow intensity of SMSO colloidal solutions in PBS, FBS, and water. All data are presented as mean values ± SD. DI, deionized.

We then characterized the luminescence properties of SMSO colloids prepared by the BID method (Fig. 2). The photoluminescence excitation and emission spectra of the SMSO colloid are identical to those of its corresponding bulk phosphor (Fig. 2, A and B). In addition, the afterglow spectrum of the SMSO colloid agrees with that of its solid-state precursor (Fig. 2C). Last, the emission half-life of the SMSO colloid (~60 s) is similar to that of its corresponding bulk phosphor (~74 s; Fig. 2D). These results represent the first example of producing stable <50-nm colloids with a strong and persistent blue afterglow matching solid-state synthesized phosphors. SMSO colloids produced from the BID method represent one of the brightest afterglow materials with blue emission and stable colloidal suspension in water (table S2), thus making it an ideal candidate to excite light-activated proteins in vivo without an external light source. Specifically, an absolute intensity measurement of an SMSO colloidal solution at 0.49 μM yields a photon emission rate of 5.25 × 10^11^ p s^−1^ cm^−2^ sr^−1^ (Fig. 2E). Furthermore, the luminescence quantum yield of SMSO colloidal solution was measured to be ~21.0% (Materials and Methods), which is higher than that of photochemical afterglow systems as previously reported (*14*, *33*).

Next, we evaluated the long-term afterglow stability of SMSO colloids in water, phosphate-buffered saline (PBS), and fetal bovine serum (FBS). The SMSO colloid exhibits the same afterglow intensity in PBS in comparison to that in water, while its lower intensity in FBS is solely attributed to the absorption of FBS at 470 nm instead of chemical instability (Fig. 2F and fig. S10). Despite different environments, the SMSO colloid exhibits chronic stability in afterglow intensity over 14 days in water, PBS, and FBS at room temperature (Fig. 2G). Together, these characterizations reveal the BID method as a preferred approach to produce high-quality colloidal solutions of nanophosphors that maintain the strong emission power of their bulk counterparts while significantly improving colloidal stability in water.

### The BID method is a generalizable approach for synthesizing colloidal solutions of nanophosphors spanning the visible spectrum

The different activation spectra of light-responsive proteins require light sources of distinct wavelengths for efficient excitation. To this end, we apply the BID method to produce colloidal solutions of nanophosphors with afterglow wavelengths spanning the entire visible spectrum. Existing bottom-up approaches usually produce afterglow nanoparticles with red to NIR emission (table S2), thus prohibiting their use as light sources to excite common light-activatable proteins such as YFP, SSFO, and psCas9. In contrast, solid-state reactions offer a wide-range combination of host materials and activating ions to yield phosphors with a strong afterglow and tunable wavelengths, yet many of which remain refractory to wet-chemical and mechanical milling methods with limited colloidal stability. For example, phosphors with strong persistent luminescence (*19*, *34*) and desirable phases (*35*) can only be produced at exceptionally high temperatures, while their color centers may be quenched by built-in stress and dislocations introduced during mechanical milling. We hypothesize that, since most solid-state phosphors have low water solubility, Eq. 2 predicts 10- to 100-nm nanoparticles produced via the BID process, thus offering a generalizable method to synthesize colloidal solutions of nanophosphors.

**Fig. 3.**
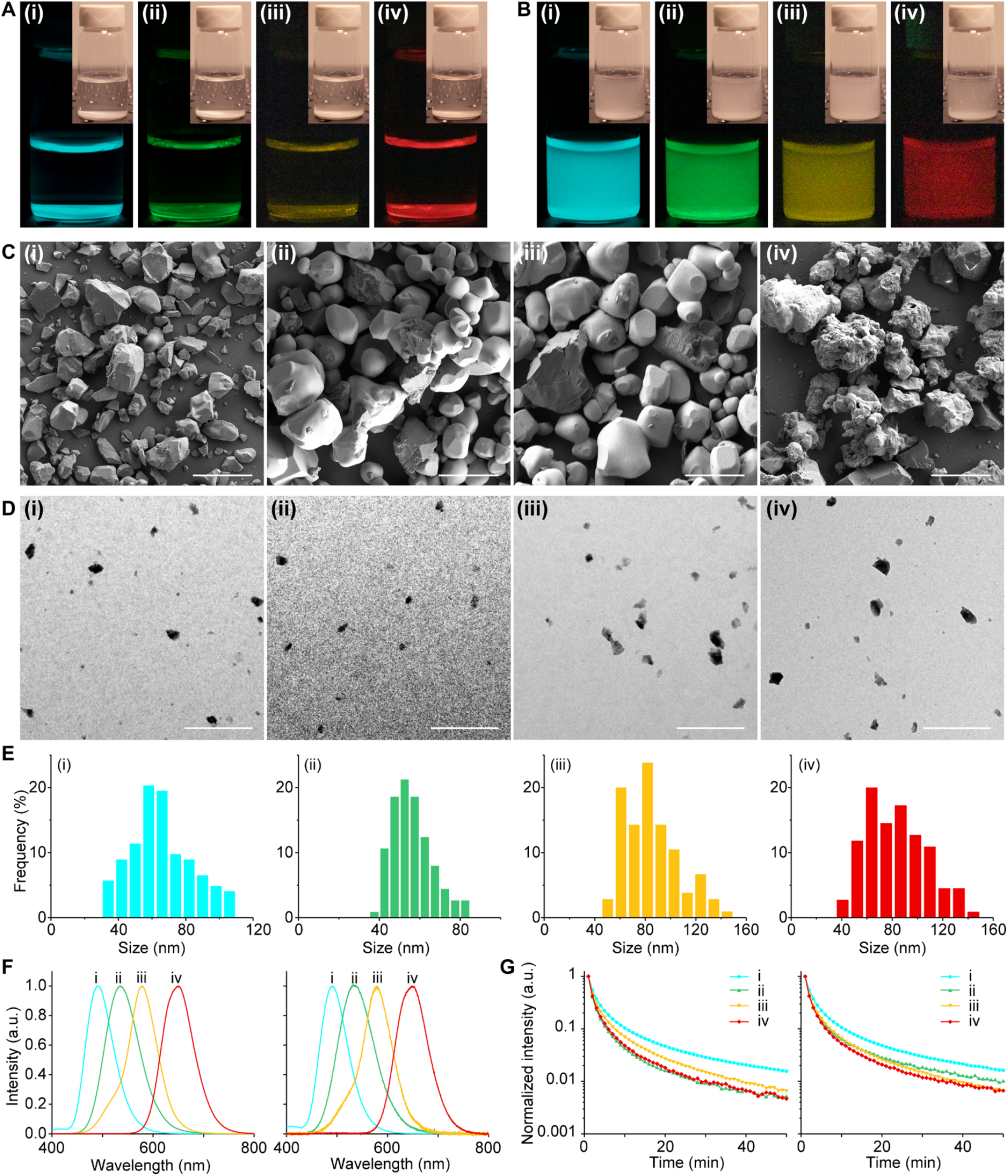
Generalizability of the BID method. (**A**) Afterglow images and their corresponding bright-field images (insets) of SAO (i), ZnS:Cu,Al (ii), ZnS:Mn (iii), and CSS (iv) bulk phosphors. (**B**) Afterglow images and their corresponding bright-field images (insets) of SAO (i), ZnS:Cu,Al (ii), ZnS:Mn (iii), and CSS (iv) nanophosphor colloids. (**C**) SEM images of SAO (i), ZnS:Cu,Al (ii), ZnS:Mn (iii), and CSS (iv) bulk phosphors. Scale bars, 50 μm. (**D**) TEM images of SAO (i), ZnS:Cu,Al (ii), ZnS:Mn (iii), and CSS (iv) nanophosphor colloids. Scale bars, 500 nm. (**E**) Histograms showing the size distributions of SAO (i), ZnS:Cu,Al (ii), ZnS:Mn (iii), and CSS (iv) nanophosphor colloids. Each histogram is based on 100 colloidal nanoparticles in the TEM images. (**F**) Afterglow spectra of SAO (i), ZnS:Cu,Al (ii), ZnS:Mn (iii), and CSS (iv) bulk phosphors (left) and colloidal nanophosphors (right). (**G**) Luminescence decay curves of SAO (i), ZnS:Cu,Al (ii), ZnS:Mn (iii), and CSS (iv) bulk phosphors (left) and colloidal nanophosphors (right).

We rationally chose a select few persistent phosphors that can only be synthesized in solid-state reactions and exhibit strong afterglow spanning the visible spectrum to demonstrate the generalizability of the BID method. These selected phosphors have very low solubility in water and do not react with water (*36*–*38*), thus fulfilling the critical size requirement to apply the BID mechanism (Eqs. 1 and 2). Specifically, Sr_4_Al_14_O_25_:Eu,Dy (SAO) exhibits strong afterglow at 490 nm and can only be synthesized at ≥1350°C. In addition, wurtzite ZnS:Cu,Al and ZnS:Mn exhibit strong afterglow at 534 and 578 nm, respectively, and their specific polymorph can only be formed at ≥1000°C. Moreover, Ca_0.85_Sr_0.15_S:Eu,Tm (CSS) represents one of the few materials with strong red afterglow at 650 nm and requires a high annealing temperature of 1100°C (see Materials and Methods). The BID method successfully produced stable colloidal solutions of nanophosphors with emission colors consistent with their bulk precursors (Fig. 3, A and B) and sizes below 100 nm (Fig. 3, C to E). Furthermore, the afterglow spectra, afterglow kinetics, and x-ray diffraction (XRD) spectra of these nanophosphor colloids are all consistent with those of their bulk precursors (Fig. 3, F and G, and fig. S11). Together, these results confirm the BID method as a generalizable approach to synthesize stable nanophosphor colloids in water, with their emission spectra spanning the entire visible spectrum (Fig. 3F and figs. S12 and S13) while preserving their desirable polymorphs and afterglow properties.

### BID-produced colloids are among the brightest reported afterglow materials after delivery in vivo

We next sought to evaluate the afterglow intensity of BID-produced nanophosphor colloids after delivery in vivo and compare it to other reported afterglow materials. Unlike their bulk precursors, BID-produced nanophosphors exhibit superior colloidal stability, thus enabling them to be delivered via conventional administration routes and act as colloidal light sources. We selected two administration methods commonly used to deliver biocompatible colloidal solutions, subcutaneous and intravenous injection (*8*, *9*, *11*, *16*). We hypothesized that the strong afterglow of BID-produced colloids enabled ultrasensitive in vivo imaging by eliminating real-time excitation and tissue autofluorescence. As a result, the afterglow of in vivo administered nanophosphor colloids should be detectable in deep tissue despite their much shorter wavelengths than other reported afterglow materials.

**Fig. 4.**
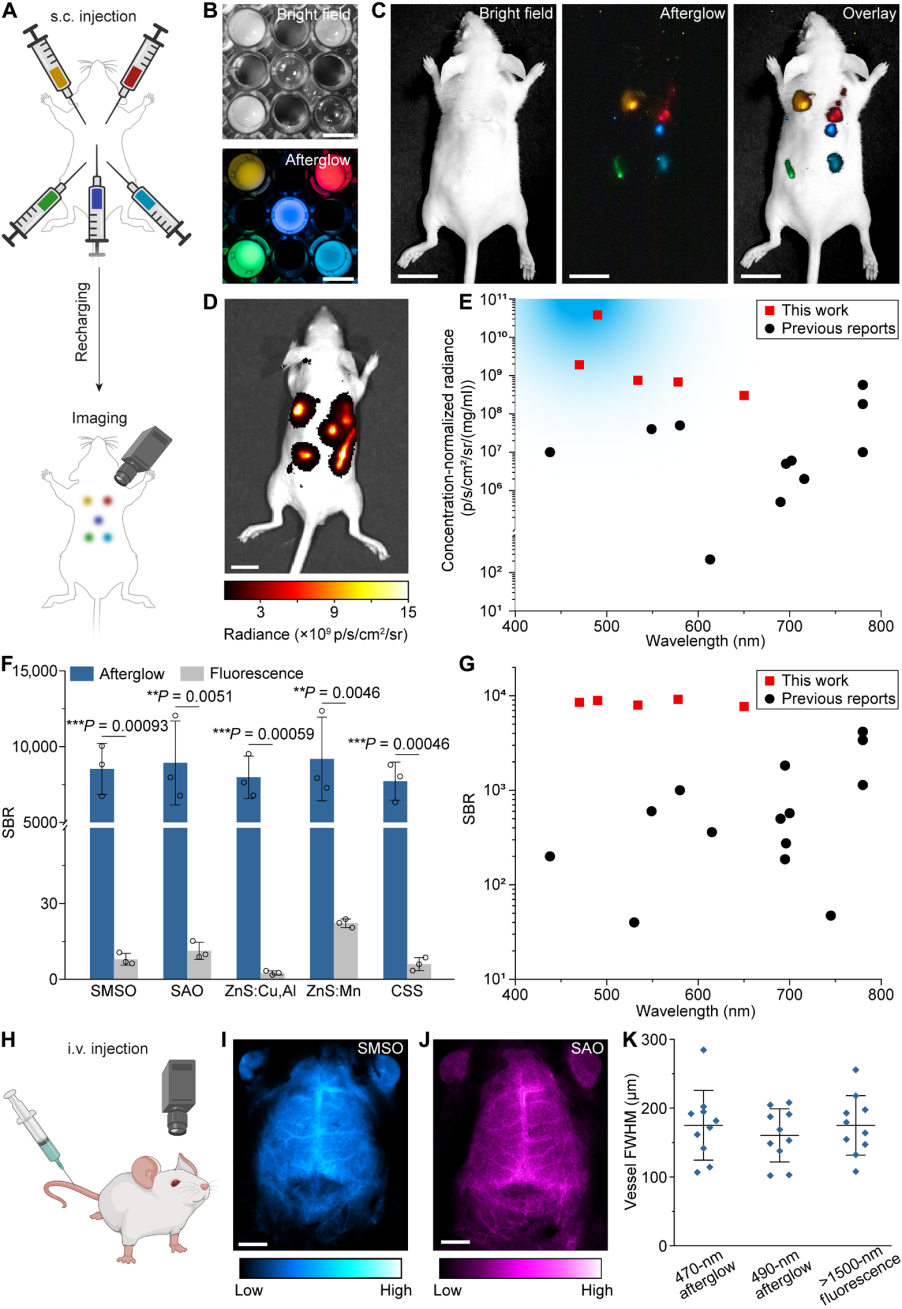
BID-produced nanophosphor colloids are among the brightest afterglow materials after delivery in vivo. (**A**) BID-produced colloids can be delivered via subcutaneous (s.c.) administration. (**B**) Bright-field (top) and afterglow (bottom) images of five colloidal solutions in a multiwell plate. (**C**) Bright-field (left), afterglow (middle), and overlay (right) images of subcutaneously administered colloidal solutions. (**D**) Afterglow image of subcutaneously injected colloids acquired using the IVIS imaging system. (**E**) Scatterplot of concentration-normalized afterglow radiance versus emission wavelength for subcutaneously injected colloids in this work and previous reports. The blue shade represents the desirable power density for activating SSFO and psCas9 in various biological applications. (**F**) Statistical analysis of the SBR for afterglow and fluorescence imaging with BID-produced nanophosphor colloids. All data are presented as mean values ± SD. *n* = 3 for all groups. **P < 0.01; ***P < 0.001. (**G**) Scatterplot of the SBR versus emission wavelength for subcutaneously injected colloidal solutions of nanophosphors in this work and previous reports. (**H**) BID-produced colloidal solutions of nanophosphors can be delivered via intravenous (i.v.) administration for brain imaging. (**I** and **J**) Transcranial afterglow images of brain vascular structures after intravenous injection of SMSO (I) and SAO (J) colloidal solutions. (**K**) Full width at half maximum (FWHM) of the smallest discernible cerebral vessels in afterglow images of this work and an NIR-II (>1500 nm) fluorescence image in (*50*) under the same level of magnification. Scale bars, 1 cm (B and D), 1.5 cm (C), and 2.5 mm (I and J).

We subcutaneously injected all five colloidal solutions of persistent nanophosphors produced above (SMSO, SAO, ZnS:Cu,Al, ZnS:Mn, and CSS) and performed multicolor afterglow imaging (Fig. 4A). The distinct afterglow colors of these colloids allowed us to resolve their spatial distribution in live mice after delivery (Fig. 4, B and C). Quantitative radiance measurements reveal strong emission intensity of all subcutaneously injected colloids despite attenuation through the skin (Fig. 4D). Notably, the mass-normalized afterglow intensity of the brightest BID-produced colloids is two orders of magnitude higher than the brightest afterglow nanomaterial reported to date in the same setting, regardless of emission wavelengths (Fig. 4E and table S2). The superiority of BID-produced nanophosphors is validated by comparing both subcutaneously injected materials in vivo and an aqueous solution of the material ex vivo (table S2). We attribute the brighter afterglow of BID-produced nanophosphors to their preservation of the optical properties of their bulk counterparts from solid-state reactions, which result in thermodynamically and kinetically more favorable formation of desirable polymorphs and point defects leading to stronger persistent luminescence. In addition, the exceptional afterglow intensity and minimal autofluorescence background enabled us to obtain a much higher signal-to-background ratio (SBR) up to 9190× than that of fluorescence imaging (<23×) with the same injected colloids (Fig. 4F). Despite their unfavorably short emission wavelengths, the SBR values of BID-produced colloids even exceeded those obtained with red and NIR afterglow materials (Fig. 4G and table S2).

Having demonstrated subcutaneous delivery of BID-produced nanophosphors, we next aimed to validate their feasibility as systemically delivered light sources via intravenous injection. Specifically, we injected the SMSO or SAO colloidal solution, which was charged before injection (Fig. 4H and fig. S14), into the mouse tail vein. Immediately after injection, transcranial afterglow imaging through the intact skull reveals that intravenously administered colloids are spatially confined in cerebral vessels. Their afterglow was sufficiently bright to be visualized even through the intact skull despite their short wavelengths (470 and 490 nm for SMSO and SAO, respectively) (Fig. 4, I and J). Line cross-sectional intensity analysis reveals spatially resolved cerebral vessels with similar widths to those imaged with fluorescence in the second NIR window (Fig. 4K and fig. S15). These images represent the first example of transcranial afterglow imaging of cerebral vessels in the mouse brain.

Furthermore, the utility of BID-produced nanophosphors as systemically delivered light sources relies on their ability to be recharged during circulation in vivo. Previous demonstration of in vivo recharging of afterglow materials usually involved in situ recharging and activation at the same site for imaging (*8*, *17*, *39*). Despite the use of red or NIR light for recharging, the efficiency of recharging still remains limited, while recharging and imaging at the same location prohibit simultaneous afterglow imaging during recharging (*1*). We hypothesize that the intrinsic circulatory system of the animal can be leveraged to efficiently recharge intravenously delivered BID-produced nanophosphors when they pass through superficial blood vessels, even with short-wavelength excitation light, at a different location in the body. To prove this hypothesis, we performed real-time afterglow imaging of the femoral artery in the mouse hindlimb while applying remote periodic recharging of the circulating afterglow colloids in superficial hepatic vessels through noninvasive transdermal photoexcitation (see Materials and Methods; fig. S16, A and B). We found that the afterglow in the femoral artery increased after every recharging pulse, with a baseline intensity ~10× higher than that without recharging at 400 s after injection (fig. S16C). These results represent the first demonstration of remote recharging of afterglow materials in vivo via the intrinsic circulatory system.

The three experiments of afterglow imaging above validated the remarkable afterglow intensity and rechargeability of BID-produced colloids in living mice. Compared to the extensively reported afterglow nanoparticles in red and NIR spectra, BID-produced colloids benefit from their strong afterglow of blue-shifted photons, which may activate many blue light–responsive proteins such as SSFO and psCas9 (see Fig. 4E, Supplementary Text, and table S1) (*3*, *13*, *31*). The demonstrated compatibility with common administration routes, especially intravenous injection, provides a means to uniformly deliver these colloidal light sources throughout the body. To prove the biosafety of intravenously delivered colloidal light sources, we performed detailed biodistribution, secretion, and toxicology studies in mice and found minimal adverse effects to the subject after systemic administration of these colloids (figs. S17 to S19). These pharmacokinetics data suggest minimal retention of systemically delivered afterglow colloids at 1 week after administration. Nonetheless, multiple injections may be performed to facilitate their long-term utility in the same animal.

### BID-produced colloidal light sources enable internal excitation of genetically encoded fluorescent reporters with greater tissue penetration

The strong afterglow of BID-produced colloids in the blue spectrum allowed us to postulate their function as an intravital light source to excite light-responsive proteins endogenously expressed in the tissue. Specifically, genetically encoded fluorescent reporters, such as fluorescent proteins and their functional derivatives (e.g., GCaMP), suffer from limited tissue penetration due to strong scattering and autofluorescence of their short excitation wavelengths. This disadvantage necessitates the invasive implantation of a cranial window or a gradient index (GRIN) lens (*40*) with limited field of view (FoV) and restrained behavior of the subject in a microscopic setup (*41*). In contrast, widefield afterglow imaging enables a much larger FoV and deeper tissue penetration by eliminating an external excitation, thus reducing scattering and autofluorescence. However, afterglow imaging has never been realized for endogenous fluorescent proteins. We hypothesized that systemically delivered colloidal light sources can enable widefield afterglow imaging of endogenously expressed fluorescent proteins with increased tissue penetration.

**Fig. 5.**
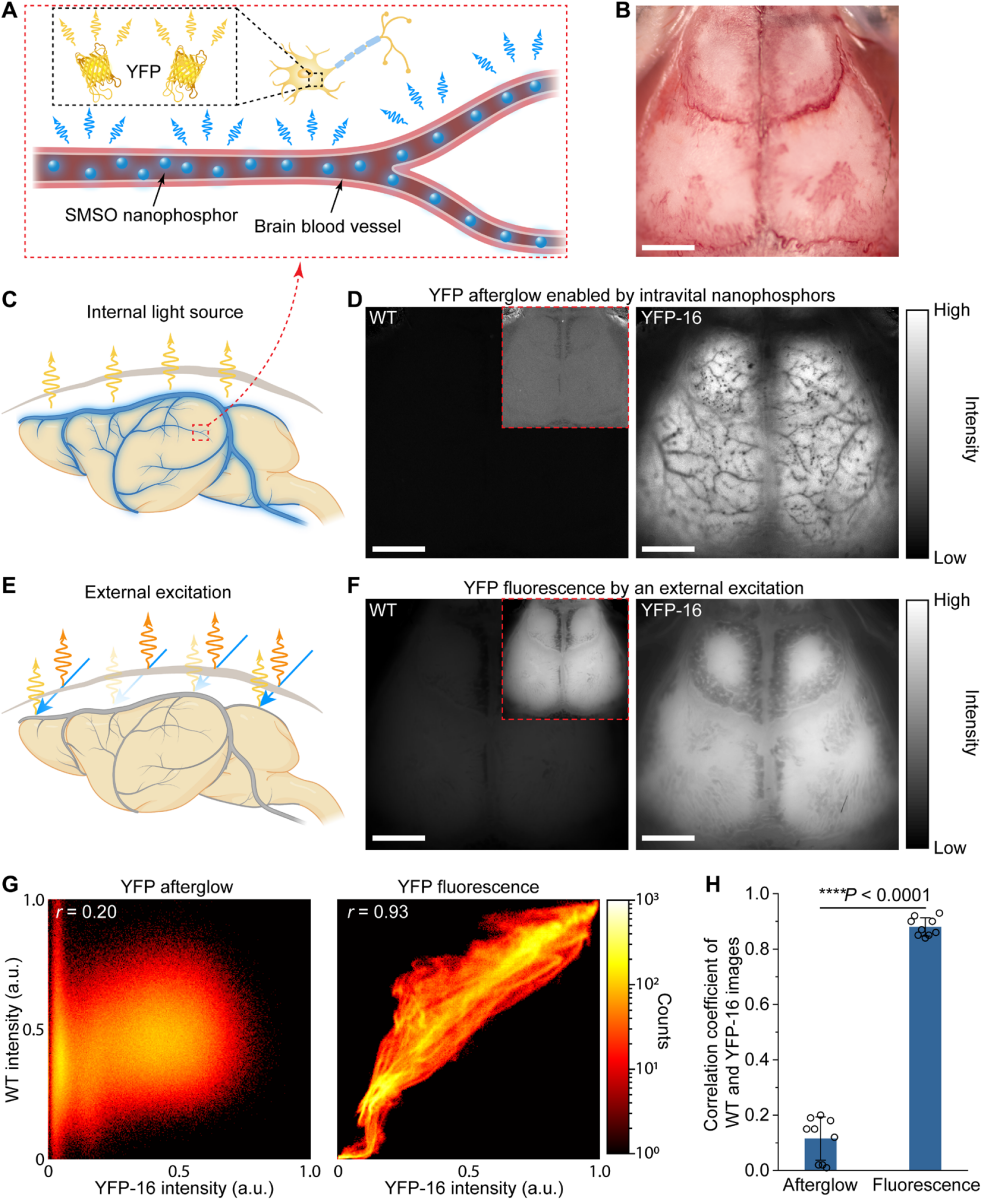
BID-produced colloids as an intravital light source for YFP imaging. (**A**) Schematic showing SMSO colloids in blood vessels as an internal light source for exciting YFP fluorescence in situ. (**B**) Photo of the mouse head with the intact skull, which is dominated by the intrinsic skull features. (**C** and **E**) Schematic of brain fluorescence imaging with an internal light source (C) or a conventional external light source (E). The blue glow represents the internal excitation light. The blue, yellow, and orange arrows represent external excitation light, YFP fluorescence, and skull autofluorescence, respectively. The fluorescence image excited by a conventional external light source (E) is contaminated by skull features because of spatially varying skull attenuation and autofluorescence. (**D** and **F**) YFP afterglow (D) or fluorescence (F) images of the wild-type (WT) (left) and YFP-16 (right) mouse brains excited by the intravenously delivered colloidal light source (D) or an external light source (F). The insets in the left panels are WT images with digitally enhanced brightness to match YFP-16 images. All images in (B), (D), and (F) were taken with the intact skull. Scale bars, 2 mm. (**G**) Intensity scatterplot of the WT and YFP-16 images under the afterglow (left) or fluorescence (right) modes. The Pearson’s correlation coefficient *r* is provided on the image. (**H**) Statistical analysis of Pearson’s correlation coefficients (indicating similarity) between the WT and YFP-16 images under the afterglow or fluorescence modes. All data are presented as mean values ± SD. ****P < 0.0001.

We set out to demonstrate widefield afterglow imaging of YFPs via BID-produced colloidal light sources. We delivered a colloidal solution of SMSO into the cerebral vessels of a transgenic mouse [B6.Cg-Tg(Thy1-YFP)16Jrs/J, YFP-16] expressing YFPs in neurons (Fig. 5A) (*42*). The spectral overlap between the afterglow spectrum of SMSO colloids and the excitation spectrum of YFP offers efficient excitation of YFP fluorescence by the afterglow of SMSO (fig. S20). We used widefield acquisition for whole-brain YFP imaging through the intact skull (Fig. 5B) to demonstrate a large FoV for potentially imaging free-behaving animals in future studies. When a traditional external excitation was applied, the excitation light was absorbed and scattered by the skull while producing autofluorescence (Fig. 5E). As a result, this process yielded epifluorescence images featuring skull structures that block YFP fluorescence from the underlying brain (Fig. 5F, right, and figs. S21 and S22). Control experiments on wild-type (WT) mice without YFP expression revealed similar patterns in the epifluorescence images (Fig. 5F, left). Pearson’s correlation analysis between fluorescence images of WT versus YFP-16 mice reveals a high Pearson’s correlation coefficient of 0.93 (Fig. 5, G, right, and H), thus confirming the origin of these features arising from the skull rather than from the underlying brain (*43*). These observations confirmed the need to remove or thin the skull in conventional YFP imaging in the mouse brain (*40*).

In contrast to the external excitation, with an internal excitation enabled by systemically delivered colloidal light sources, YFP fluorescence was collected in the afterglow mode (see Materials and Methods; Fig. 5C). The resulting YFP afterglow image of the same mouse brain only revealed the spatial distribution of YFPs in the brain (i.e., in the brain parenchyma while excluding cerebral vessels) without any interference from the skull (Fig. 5D, right, and fig. S21). Unlike epifluorescence imaging, afterglow images of WT versus YFP mouse brain exhibited dissimilar patterns (Fig. 5D). A low Pearson’s correlation coefficient of 0.20 suggests minimal feature colocalization between the WT versus YFP-16 mouse brain in the afterglow mode (Fig. 5, G, left, and H). These results demonstrate the unique ability of afterglow imaging to reveal deep-tissue features unresolvable by conventional fluorescence imaging with the intact skull. In summary, BID-produced colloidal light sources enabled widefield afterglow imaging of YFPs with minimal autofluorescence and scattering background from overlying tissues. Therefore, in vivo widefield afterglow imaging offers a much less invasive approach than conventional microscopy through a cranial window for imaging genetically encoded fluorescent reporters in the mouse brain.

## DISCUSSION

Here, we report a generalizable method for producing stable colloidal solutions of nanophosphors from their solid-state bulk materials with exceptionally bright luminescence (up to 5.25 × 10^11^ p s^−1^ cm^−2^ sr^−1^), tunable emission wavelengths down to 470 nm, and small sizes (down to 20 nm). This method was enabled by the BID process, which has been found in many biominerals such as tooth enamel and avian eggshells to afford exquisite control of complex nanostructures (*25*, *26*). Colloids produced by this approach exhibited the highest SBR for in vivo imaging among reported materials, yielding the first example of transcranial afterglow imaging of cerebral vessels in the mouse brain. Because of their strong emission intensity and colloidal stability in water, BID-produced colloidal light sources provide internal excitation of and enable afterglow imaging of genetically encoded fluorescent reporters in a mouse brain through the intact skull.

Compared to previously reported afterglow materials, the BID approach and BID-produced colloids have three unique advantages that enable their use as an internal light source. First, the BID approach can be applied to a wide range of solid-state phosphors to produce tunable emission down to 470 nm. These inorganic phosphors usually require specific polymorphs and dopant ions incompatible with conventional sol-gel synthesis and mechanical ball milling. As a result, the BID approach contrasts strongly with the limited range of conventional afterglow materials including ZnGa_2_O_4_:Cr^3+^ (695 nm), semiconducting polymers (780 nm), and rare-earth doped NaLnF_4_ nanoparticles (>1000 nm) (*8*, *9*, *16*). Second, the BID approach preserves the crystallinity and emission intensity of solid-state phosphors in synthesized colloidal nanophosphors. This preservation is owing to a mild top-down process that produces the colloids, in strong contrast with harsh mechanical milling that usually yields mechanical and dislocation-induced quenching (*23*, *24*). Using the in vivo intensity after subcutaneous injection as a metric for comparison, BID-produced colloids [~3.8 × 10^10^ p s^−1^ cm^−2^ sr^−1^ (mg/ml)^−1^; table S2] are two orders of magnitude higher than the brightest afterglow material reported previously [~10^8^ p s^−1^ cm^−2^ sr^−1^ (mg/ml)^−1^; table S2]. Furthermore, in an ex vivo solution, BID-produced colloids are five orders of magnitude brighter [6.2 × 10^11^ p s^−1^ cm^−2^ sr^−1^ (mg/ml)^−1^; table S2] than the brightest afterglow nanoparticles produced by other methods such as ball milling and grinding [2 × 10^6^ p s^−1^ cm^−2^ sr^−1^ (mg/ml)^−1^; table S2]. The high quality of BID-produced colloids is also evidenced by remarkable stability over 1000 repeated recharging and emission cycles (fig. S23) and superior resistance against photobleaching even after prolonged exposure to strong excitation light (fig. S24), in contrast to rapid decay of organic afterglow materials (*9*, *11*). Third, the colloidal stability of BID-produced nanophosphors in water yields sufficient biosafety for them to be used as systemically delivered light sources in live mice (Fig. 5). The demonstrated ability of transcranial afterglow imaging of YFPs thus offers a minimally invasive approach to image and modulate gene expression and neural activity in the mouse brain without any cranial windows or implanted GRIN lenses.

Furthermore, the BID method also enables unique opportunities for synthesizing stable colloidal suspensions of other functional materials for biomedical applications. Specifically, besides persistent phosphors, other functional materials that can only be synthesized via high-temperature solid-state reactions or annealing can also be processed via the BID method to yield stable colloidal suspensions in water. Many ceramic materials, such as mechanoluminescent ZnS (*44*), piezoelectric LiTaO_3_ (*45*), and ferroelectric materials LiNbO_3_ (*46*), can only be produced in the bulk and are thus insoluble in water. Nonetheless, we can leverage the same BID principle to produce corresponding stable colloids while preserving the structure and properties of their bulk counterparts for biological applications, such as in vivo fluorescence imaging (*41*), sono-optogenetics (*44*), ultrasound and magnetic neuromodulation (*47*), and flexible optoelectronics (*48*).

## MATERIALS AND METHODS

### Chemicals

Strontium carbonate (SrCO_3_, ≥99.9%), silicon dioxide (SiO_2_, 99.5%), boric acid (H_3_BO_3_, ≥99.5%), europium oxide (Eu_2_O_3_, 99.99%), dysprosium oxide (Dy_2_O_3_, 99.99%), magnesium carbonate hydroxide pentahydrate [(MgCO_3_)_4_·Mg(OH)_2_·5H_2_O, 99.5%], aluminum oxide (Al_2_O_3_, 99.99%), zinc sulfide (ZnS, 99.99%), copper(II) acetylacetonate (≥99.9%), manganese(II) carbonate (MnCO_3_, ≥99.9%), sulfur (99.98%), calcium carbonate (CaCO_3_, ≥99.95%), thulium(III) acetate hydrate (99.9%), sodium citrate dihydrate (≥99%), strontium chloride (SrCl_2_, ≥99.99%), magnesium chloride (MgCl_2_, 99.9%), and citric acid (≥99.5%) were purchased from Sigma-Aldrich Inc. Ethanol, FBS, hydrochloric acid, nitric acid (HNO_3_), 1× PBS, paraformaldehyde, and *N*,*N*-dimethylformamide (DMF) were purchased from Thermo Fisher Scientific. Methoxy(polyethylene glycol)-silane (mPEG-silane; 20 kDa) was purchased from Jenkem Technology. All chemicals were used as purchased.

### Synthesis of SMSO bulk phosphor and colloidal nanophosphor

SMSO nanophosphor colloid was prepared by a two-step method. First, SMSO bulk phosphor was synthesized via a high-temperature solid-state chemical reaction. In this reaction, 1166.28 mg of SrCO_3_ (7.9 mmol), 388.52 mg of (MgCO_3_)_4_·Mg(OH)_2_·5H_2_O (0.8 mmol), 480.64 mg of SiO_2_ (8 mmol), 4.2 mg of Eu_2_O_3_ (0.012 mmol), 14.92 mg of Dy_2_O_3_ (0.04 mmol), and 14.83 mg of H_3_BO_3_ (0.24 mmol) were added into an agate mortar and then ground by a pestle for 1 hour. Then, the mixed precursors were annealed at 1050°C for 2 hours under a reduction atmosphere of 5% H_2_ in Ar. After cooling to room temperature, the as-prepared SMSO bulk materials were ball-milled using zirconia beads in a high-energy ball mill (8000D Mixer/Mill, SPEX SamplePrep, Metuchen, NJ) for 30 min. In the second step, sodium citrate buffer (0.08 M, pH 6) was prepared as the BID solution. Subsequently, the ball-milled SMSO particles (200 mg) and 24 ml of sodium citrate buffer were added into a flask and stirred at 80°C for 72 hours, during which the pH of the solution quickly increased to and stabilized at ~10. Final SMSO nanophosphor colloid was obtained by centrifugation at 1000 rpm (Sorvall Legend X1R Centrifuge; Thermo Fisher Scientific, Waltham, MA) for 10 min to remove large parent particles.

### Synthesis of SAO bulk phosphor and colloidal nanophosphor

SAO bulk phosphor was prepared by a conventional solid-state reaction. In total, 1476 mg of SrCO_3_ (10 mmol), 1784 mg of Al_2_O_3_ (17.5 mmol), 26 mg of Eu_2_O_3_ (0.075 mmol), and 46 mg of Dy_2_O_3_ (0.125 mmol) were weighed and thoroughly ground in an agate mortar for 1 hour. The mixed powder was annealed at 1350°C for 2 hours under the reduction atmosphere of 5% H_2_ in Ar. After cooling to room temperature, the prepared SAO bulk powder was milled using zirconia beads in a high-energy ball mill for 30 min. The same BID procedure as in the preparation of the SMSO colloid was followed to synthesize the SAO colloid.

### Synthesis of ZnS:Cu,Al bulk phosphor and colloidal nanophosphor

ZnS:Cu,Al bulk phosphor was prepared by a conventional solid-state reaction. First, 2.62 mg of copper acetylacetonate (0.01 mmol) was dissolved in 1 ml of chloroform to prepare a copper precursor solution (0.01 M). Subsequently, 0.98 g of ZnS (10 mmol) and 3 mg of Al_2_O_3_ (0.03 mmol) were weighed and put into an agate mortar. Meanwhile, 10 μl of the copper precursor solution and 6 mg of H_3_BO_3_ as the flux were added into the agate mortar and thoroughly ground for 1 hour. Last, the mixed powder was annealed at 1100°C for 2 hours under a reduction atmosphere of 5% H_2_ in Ar. After cooling to room temperature, the prepared ZnS:Cu,Al powder was ball-milled using zirconia beads in a high-energy ball mill for 30 min. The same BID procedure as in the preparation of the SMSO colloid was followed to synthesize the ZnS:Cu,Al colloid.

### Synthesis of ZnS:Mn bulk phosphor and colloidal nanophosphor

ZnS:Mn bulk phosphor was prepared by a conventional solid-state reaction. In total, 0.98 g of ZnS (10 mmol), 1.15 mg of MnCO_3_ (0.01 mmol), and 6 mg of H_3_BO_3_ as the flux were weighed and ground in an agate mortar for 1 hour. The mixed powder was then annealed at 1100°C for 2 hours under a reduction atmosphere of 5% H_2_ in Ar. After cooling to room temperature, the prepared ZnS:Mn powder was ball-milled using zirconia beads in a high-energy ball mill for 30 min. The same BID procedure as in the preparation of the SMSO colloid was followed to synthesize the ZnS:Mn colloid.

### Synthesis of CSS phosphor and colloidal nanophosphor

CSS bulk phosphor was prepared by a conventional solid-state reaction. In total, 1 g of CaCO_3_ (10 mmol), 250 mg of SrCO_3_ (1.7 mmol), 0.7 mg of Eu_2_O_3_ (0.004 mmol), 4.8 mg of thulium(III) acetate hydrate (0.014 mmol), and 6 mg of H_3_BO_3_ (0.1 mmol) as the flux were weighed and ground in an agate mortar for 1 hour. Then, 1283 mg of sulfur (40 mmol) was added into the mixed powder and annealed at 1100°C for 1 hour using activated charcoal to provide a reducing atmosphere. After cooling to room temperature, the prepared CSS powder was ball-milled using zirconia beads in a high-energy ball mill for 30 min. Subsequently, the ball-milled CSS particles (200 mg) and 24 ml of the BID solution were added into a flask and stirred at room temperature for 2 hours. Final CSS colloid was obtained by centrifugation at 1000 rpm (Sorvall Legend X1R Centrifuge; Thermo Fisher Scientific, Waltham, MA) for 10 min to remove large parent particles.

### Surface functionalization of nanophosphor colloids

For in vivo applications, the as-prepared colloids were functionalized with mPEG-silane (20 kDa). First, the SMSO colloid was transferred into a cellulose dialysis tubing (molecular weight cutoff = 30 kDa) and dialyzed against water for 2 days to remove excess citrate ions. Subsequently, surface hydroxylation was performed on the surface of the SMSO colloid. Specifically, a 20-μl solution of sodium hydroxide (10 M) was added into 10 ml of SMSO colloid (67 nM) and sonicated for 1 hour at room temperature (*49*). After surface hydroxylation, the SMSO colloid was washed with water and anhydrous DMF three times in each solvent and dispersed in DMF. Last, 40 mg of mPEG-silane (20 kDa) was added into 10 ml of the SMSO DMF solution and sonicated at 50°C for 4 hours. The PEGylated-SMSO colloid was washed with pure DMF and water three times in each solvent and then dispersed in PBS for in vivo experiments. The same PEGylation procedure was followed to prepare the solutions containing SAO, ZnS:Cu,Al, ZnS:Mn, and CSS colloids.

### Characterizations

XRD patterns were acquired by using a diffractometer (PANalytical Empyrean, Malvern Panalytical Ltd., Malvern, UK) with Cu Kα (λ = 1.540598 Å) radiation at a 45-kV tube voltage and a 40-mA tube current. The transmission electron microscope (TEM) images of the samples were captured on a Field Electron and Ion Company Tecnai TEM (FEI Company, Hillsboro, OR) equipped with a charge-coupled device (CCD) camera. SEM images were recorded by an Apreo S LoVac SEM (Thermo Fisher Scientific, Waltham, MA). FTIR spectroscopy was performed using a Nicolet iS50 FT/IR spectrometer (Thermo Fisher Scientific, Waltham, MA) on dried powder samples. Absorption spectra were measured with the Evolution 350 UV-Vis Spectrophotometer (Thermo Fisher Scientific, Waltham, MA). For the UV-vis measurement of the silica-citrate complex, a Savitzky-Golay filter with a window size of 10 is applied because of its low absorption. All other UV-vis absorption spectra are displayed as is. Photoluminescence spectra of all samples were measured with a Horiba FluoroLog Fluorometer spectrophotometer (HORIBA Ltd., Kyoto, Japan) in a 1-cm quartz cuvette. In photoluminescence measurements, the excitation wavelength was 380 nm and the measured range of photoluminescence was 400 to 700 nm. Elemental analyses of the samples were characterized by an inductively coupled plasma mass spectrometer (ICP-MS; Thermo Scientific X-SERIES II Quadrupole, Thermo Fisher Scientific, Waltham, MA). NMR spectra were collected on a 500-MHz Varian Inova NMR spectrometer (Varian Inc., Palo Alto, CA). The Brunauer-Emmett-Teller (BET) isotherm was measured with a surface area and pore size analyzer (Anton-Paar NovaTouch, Boynton Beach, FL).

### Determining molar concentrations of nanophosphor colloids

The molar concentration of each colloid was calculated with the Beer-Lambert law (*A =* Ɛ*lc*), in which *A* is the absorbance, Ɛ is the extinction coefficient per mole of colloidal nanoparticles per liter (liter mol^−1^ cm^−1^), *l* is the optical path length (cm), and *c* is the molar concentration (M) of the colloidal nanoparticles of the same sample. Specifically, Ɛ was determined by linear fitting of the measured absorbance at 400 nm for a specific colloid at three concentrations against their concentrations determined by ICP-MS measurements.

### Real-time confocal fluorescence microscopy of the BID process

The BID process of SMSO particles in the sodium citrate buffer was characterized with real-time fluorescence imaging, which was performed on an LSM 980 confocal microscope (Carl Zeiss, Oberkochen, Germany) using a 63× oil-immersion Plan-APO objective (numerical aperture: 1.4) with an excitation wavelength of 405 nm and an exposure time of 1.62 s per image. The filter set consisted of an excitation filter at 405 nm, a beam splitter at 435 nm, and an emission filter at 465 nm. A z-stack of 31 slices with an interval of 0.38 μm (pinhole size: 1.83 airy units) was acquired per time frame to account for the vertical drifting of SMSO particles.

### The CC technique

Dissolution experiments with the CC technique were performed by following previously reported protocols (*25*, *27*). Specifically, all reactions were carried out in magnetically stirred three-neck round-bottom flasks. Undersaturated reaction solutions (60 ml) were prepared by mixing 360 μl of SrCl_2_ (1 M) and 180 μl of MgCl_2_ (1 M) solutions with the sodium citrate buffer (0.08 M) such that [Mg^2+^] = 3 mM and [Sr^2+^] = 6 mM. The pH was adjusted to the desired value with either hydrochloric acid (12.1 M) or sodium hydroxide (10 M). The undersaturated solution was then heated and kept at 90°C in a silicon oil bath during the reaction. The CC dissolution reaction was initiated by introducing bulk SMSO particles (100 mg) with a specific surface area of 43,850 cm^2^/g (fig. S6). During the reaction, the pH of the solution was constantly monitored using a benchtop pH meter (SevenCompact S230; Mettler Toledo, Greifensee, Switzerland), which provided feedback control for the titrant [0.08 M citrate buffer (pH 1.75)] to keep the pH within ±0.1 of the desired value. The composition and concentration of the titrant were calculated to ensure that [MgCit^−^], [SrCit^−^], [HCit^2−^], and [Cit^3−^] all stayed constant during the dissolution reaction if the pH remained constant according to the equation in fig. S1. Because of the unknown stoichiometry of the silica-citrate complex, we did not control its concentration at a constant, and we argue that the potential formation of silica clusters contributes a unity activity (e.g., independent of its concentration) to the chemical equilibrium.

### Proving kinetically preserved dissolution to produce nanophosphor colloids

In this kinetically preserved dissolution experiment, 1000 mg of ball-milled SMSO particles and 120 ml of sodium citrate buffer [0.08 M (pH 6)] were added into a flask and stirred at 80°C for 10 min. Then, the SMSO suspension was centrifuged at 1000 rpm for 10 min to remove large parent particles and at 8000 rpm for 20 min to separate nanoparticles. The supernatant, which contained an undersaturated solution for SMSO, was collected and equally divided into two parts: Solution 1 was added with the SMSO nanophosphor colloid and kept stirring at 80°C for 3 days. A bright-field image, an image showing the Tyndall effect with a 632.8-nm HeNe laser beam (Thorlabs), and an afterglow image were taken at days 0 and 3 for this mixture (Fig. 1J, bottom row). Meanwhile, the UV-vis spectrum of this mixture was measured at days 0 and 3 (fig. S8). Solution 2 was added with SMSO bulk precursor particles and kept stirring at 80°C for 3 days. The same images were taken at day 0 for the mixture of solution 2 and at day 3 for the mixture of solution 2 after passing 1000 rpm for 10 min (Fig. 1J, top row).

### Spectral characterizations of persistent luminescence

Persistent luminescence spectra of bulk phosphors and colloidal nanophosphors were acquired using a fiber-coupled spectrometer (OCEAN-HDX-VIS-NIR; Ocean Optics, Orlando, FL) that measures the whole spectrum in the range of 350 to 900 nm spontaneously. A polydimethylsiloxane (PDMS) phantom containing a specific phosphor or nanophosphor colloid was used as the sample for spectral characterizations. Specifically, the PDMS phantom was charged by a 365-nm light-emitting diode (LED; SOLIS-365C, Thorlabs, Newton, NJ) at 5.7 mW/mm^2^ for 10 s. The persistent luminescence spectrum was acquired immediately after the charging light was turned off. Averaging over multiple measurements was applied as needed to reduce the noise of the spectrum.

### Time-resolved intensity measurements of persistent luminescence

PDMS phantoms as prepared above were charged for 10 s by a 365-nm LED at 0.13 mW/mm^2^, and a photomultiplier tube (PMT1001; Thorlabs, Newton, NJ) was put in the close vicinity of the phantom to collect the persistent luminescence after the charging light was turned off. The output voltage from the PMT, which exhibits a linear dependence on light intensity, was then collected with a multifunction input/output (I/O) device (NI USB-6221, National Instruments, Austin, TX).

### Luminescence quantum yield measurement

The luminescence quantum yield measurement was conducted by following protocols established in previous reports (*14*, *33*, *50*). Specifically, the SMSO nanophosphor colloid was excited by a collimated 365-nm light beam coupled from an LED (M365LP1, Thorlabs, Newton, NJ). An integrating sphere (IS200, Thorlabs, Newton, NJ) and a nonscanning fiber-coupled spectrometer (OCEAN-HDX-VIS-NIR; Ocean Optics, Orlando, FL) were used to redirect and collect the excitation and emission light simultaneously both during and after the recharging. The absorbed photons were measured by replacing the SMSO colloid with water and repeating the above procedure. The luminescence quantum yield was then calculated as follows

$$\text{QY}=\frac{\text{Photons emitted}}{\text{Photons absorbed}}=\frac{\int_{0}^{t_{\text{Lum}}}I_{\text{Em}}\;\mathrm{dt}}{(I_{\text{Ex}\_\text{ref}}-I_{\text{Ex}\_\text{SMSO}})\;t_{\text{Ex}}}$$

where QY is the luminescence quantum yield; *I*_Em_ is the emission light intensity; *I*_Ex_ref_ and *I*_Ex_SMSO_ are the excitation light intensity in the presence of water or SMSO nanophosphor colloid, respectively; and *t*_Lum_ and *t*_Ex_ are the duration of luminescence and excitation light, respectively.

### Repetitive recharging stability assessment

PDMS phantoms as prepared above were charged by a 365-nm LED at 1 mW/mm^2^ for 100 ms, followed by image acquisition using a scientific complementary metal-oxide semiconductor (CMOS) camera (CS165MU, Thorlabs, Newton, NJ) with 1-Hz frame rate for five frames, resulting in a recharging duty cycle of 2%. The same recharging and image acquisition cycle was repeated for 1000 times to assess the repetitive recharging stability of BID-produced nanophosphor.

### Photobleaching resistance assessment

PDMS phantoms as prepared above were charged by a 365-nm LED at 1 mW/mm^2^ for 100 ms, followed by image acquisition using a scientific CMOS camera. Afterward, the phantoms were bleached by 1 mW/mm^2^ for 24 hours continuously. Then, the same recharging and image acquisition procedure was repeated, and the afterglow intensities of the phantoms were normalized against those before photobleaching.

### Vertebrate animal subjects

Adult (20 to 30 g) BALB/cJ mice (male, 8 weeks old, The Jackson Laboratory, Bar Harbor, ME), J:Nu nude mice (male, 6 weeks old, The Jackson Laboratory, Bar Harbor, ME), C57BL/6J mice (male, 6 weeks old, The Jackson Laboratory, Bar Harbor, ME), and B6.Cg-Tg(Thy1-YFP)16Jrs/J mice (male, 6 weeks old, The Jackson Laboratory, Bar Harbor, ME) were the vertebrate animal subjects used in this study. All procedures performed on mice were approved by Stanford University’s Administrative Panel on Laboratory Animal Care (APLAC). The animal care and use programs at Stanford University meet the requirements of all federal and state regulations governing the humane care and use of laboratory animals, including the U.S. Department of Agriculture Animal Welfare Act, and PHS Policy on Humane Care and Use of Laboratory Animals. The laboratory animal care program at Stanford is accredited by the Association for the Assessment and Accreditation of Laboratory Animal Care (AAALAC International). Animals were group-housed on a 12:12-hour light:dark cycle in the Stanford University’s Veterinary Service Center (VSC) and fed with food and water ad libitum as appropriate.

### Persistent luminescence imaging of subcutaneously injected nanophosphor colloids

Nude mice were anesthetized by intraperitoneal injection of ketamine (80 mg/kg) (KetaVed, Vedco Inc., St. Joseph, MO) and dexdomitor (1 mg/kg) (Dexmedesed, Dechra Veterinary Products, Overland Park, KS). To maintain the body temperature and prevent hypothermia, a homeothermic blanket (Harvard Apparatus, Holliston, MA) was set to 37°C and placed underneath the anesthetized mouse. A blackout fabric (BK5; Thorlabs, Newton, NJ) was put underneath the mice to reduce the background reflection when taking the image. Fifty microliters of colloidal solutions (146 nM SMSO, 4 nM SAO, 80 nM ZnS:Cu,Al, 31 nM ZnS:Mn, and 81 nM CSS) were then injected subcutaneously at five different locations under the dorsal skin as indicated by Fig. 4A. The colloids were then charged by a 365-nm LED at 0.5 mW/mm^2^ for 5 s before image acquisition. Color images in Fig. 4C were acquired using the abovementioned color digital camera with an ISO of 12,800 and an exposure time of 10 s. The image in Fig. 4D was acquired using an IVIS Spectrum small animal imaging system (Spectral Instruments Imaging, Tucson, AZ), with an exposure time of 0.5 s at 10 s after the cease of the charging light.

### Persistent luminescence brain vascular imaging

BALB/cJ mice were anesthetized using the abovementioned ketamine/dexdomitor cocktail and placed on the homeothermic blanket set to 37°C. Hair removal lotion (Nair, Church & Dwight, Ewing, NJ) was used for depilation of the mouse head, and iodophor was applied to sterilize the depilated scalp skin. Incision and its elongation were made by surgical scissors to expose the cranial bone. The blackout fabric was placed underneath the mouse as mentioned above. Two hundred microliters of precharged nanophosphor colloids dispersed in 1× PBS (493 nM SMSO and 8 nM SAO) was then delivered into the bloodstream through tail-vein injection, and the persistent luminescence image was acquired immediately after the injection using an electron-multiplying CCD (EMCCD; iXon Ultra 888, Andor Technology, Belfast, UK) with a 30-s exposure time.

### Remote recharging and afterglow imaging of the femoral artery

Nude mice were anesthetized using the abovementioned ketamine/dexdomitor cocktail and placed on the homeothermic blanket set to 37°C. Incision and its elongation on the hindlimb skin were made by surgical scissors to expose the femoral artery. The blackout fabric was placed underneath the mouse as mentioned above. Two hundred microliters of precharged nanophosphor colloids dispersed in 1× PBS (8 nM SAO) was then injected intravenously. Five seconds after the injection, 15 frames of afterglow images (~31.5 s in total) were acquired without recharging using the EMCCD with 2-s exposure time and 0.1-s interframe latency. Subsequently, a collimated 365-nm light beam (1 mW/mm^2^) coupled from an LED was turned on for 2 s to recharge the circulating nanophosphors in the hepatic vessels in the liver region. Ten frames of afterglow images (~21 s in total) were acquired using the EMCCD with the same imaging parameters following each recharging pulse. This remote recharging and afterglow imaging cycle was repeated for 10 periods. See fig. S16A for more information.

### Afterglow imaging of YFPs in the mouse brain

After delivery of an SMSO colloidal solution (493 nM), the scalp was removed to expose the skull, and the SMSO colloid in the brain blood vessels was charged by a 365-nm LED at 0.5 mW/mm^2^ for 5 s before image acquisition using the EMCCD with 30-s exposure time. Two afterglow images were collected using a 482-nm band-pass (482 BP) filter and a 550-nm long-pass (550 LP) filter, respectively. To account for the spatial variation of the light source inside blood vessels and get the YFP afterglow images in Fig. 5D, we subtracted the background of the 550 LP and 482 BP images and then performed flat-field correction by normalizing the 550 LP image against the 482 BP image.

### In vivo biodistribution study

ICP-MS was used to study the biodistribution of systemically delivered SMSO colloids in vivo. Six BALB/cJ mice intravenously injected with 200 μl of SMSO colloid dispersed in 1× PBS (493 nM) were divided into two groups (*n* = 3 for each group). Then, the two groups of mice were sacrificed at 24 and 168 hours after injection, respectively, and main organs including the heart, liver, spleen, lung, kidneys, and brain were collected. Organs were weighed and dissolved in 35% HNO_3_ at 70°C overnight. The digested HNO_3_ solutions were diluted 20 times by water and measured by ICP-MS. The percent injected dose per gram of tissue (%ID/gram) in each organ was obtained by normalizing the amount of nanophosphors retained in the organ against both the initial injected dose and the organ mass.

### Histological study of potential tissue damage after colloid injection

Three BALB/cJ mice intravenously injected with 200 μl of SMSO colloids dispersed in 1× PBS (493 nM) were sacrificed at 30 days after injection. In addition, three uninjected mice were sacrificed as the control group. Major organs (brain, heart, liver, spleen, lungs, and kidneys) were harvested and fixed in 4% paraformaldehyde. After 48-hour fixation, these organs were embedded in paraffin, followed by sectioning at 10-μm slices. These organ slices were stained with hematoxylin and eosin (H&E) and imaged using a microscope (Leica DM2700 M, Wetzlar, Germany).

### Metabolic study for quantifying excretion of injected nanophosphor colloids

Three BALB/cJ mice were individually housed in a metabolic cage after being intravenously injected with 200 μl of SMSO colloids dispersed in 1× PBS (493 nM). Feces and urine samples were collected over 7 days. These samples were weighed and dissolved in 35% HNO_3_ at 70°C overnight and then measured by ICP-MS. The %ID/gram value in each sample was obtained by normalizing the amount of nanophosphors detected in the sample against both the initial injected dose and the excreta mass.

### Statistical analysis

The variance in SBR of afterglow or fluorescence imaging (Fig. 4F) and Pearson’s correlation coefficients of WT and YFP-16 afterglow or fluorescence images (Fig. 5H) was calculated, by which the pooled SD among each experimental group was determined. Comparisons between experimental groups were made using one-way analysis of variance (ANOVA) without normality assumption given its reasonable tolerance of violations to normal distribution (*51*). *P* values of less than 0.05 were considered statistically significant. The values of *N*, *F*, and *P* are provided in the figure captions.

## Supplementary Materials

### This PDF file includes:

Supplementary Text Figs. S1 to S24 Tables S1 and S2 References
