# Supplementary material for: A biomineral-inspired approach of synthesizing colloidal persistent phosphors as a multicolor, intravital light source
Source: Sci Adv. 2022 Jul 29;8(30):eabo6743. doi: 10.1126/sciadv.abo6743 (PMC9337768; doi:10.1126/sciadv.abo6743)
Supplement: Supplementary file 1 — Supplementary Text Figs. S1 to S24 Tables S1 and S2 References [file sciadv.abo6743_sm.pdf]

Science Advances
AAAS

Supplementary Materials for

A biomineral-inspired approach of synthesizing colloidal persistent phosphors as a multicolor, intravital light source

Fan Yang *et al.*

Corresponding author: Guosong Hong, guosongh@stanford.edu

Sci. Adv. **8**, eabo6743 (2022)
DOI: 10.1126/sciadv.abo6743

This PDF file includes:

Supplementary Text
Figs. S1 to S24
Tables S1 and S2
References

Supplementary Text

Estimation of the ratio of bulk SMSO precursor particles to kinetically preserved SMSO nanoparticles

In compliance with the kinetic preservation model of the BID method, two plausible but not mutually exclusive models exist to explain the origin of produced nanoparticles. Specifically, each micron-sized particle may be etched to a single nanoparticle with its size resistant to further dissolution. Alternatively, during dissolution of a micron particle, it may "shed" multiple nanoparticles from its surface as the pit expands. To this end, we calculated the final concentration of nanoparticles after the BID process as follows:

In a typical BID experiment described in Materials and Methods, an overall yield of 13% was found for SMSO nanophosphors, and the initial particle concentration of bulk SMSO precursor particles and kinetically preserved SMSO nanoparticles were 5.59 × 10^6 and 9.08 × 10^{12} particles/mL, respectively, based on UV-Vis absorption measurement and their respective sizes. This result indicates that on average, every single micron-sized SMSO particle produces ~ 10^6 nanoparticles during the kinetically preserved dissolution process. Therefore, although the first model cannot be excluded, the second model dominates the BID process to produce many small nanoparticles from their parent micron-sized particles. Further evidence is found from a close-up view of the large particles during dissolution, revealing many small nanoparticles loosely attached to the surfaces (Fig. 1E, the far right panel). A similar phenomenon has been observed during demineralization of tooth enamel, in which nanosized particles were released into the solution by fluid diffusion flux (*25*).

Estimation of highest achievable afterglow power density *in vivo* after intravenous delivery for biological applications

We first estimate the afterglow emission power per unit mass of nanophosphors ($\frac{P}{m_{BID}}$) using the *ex vivo* radiance measurement data (i.e., Fig. 2E) as follows:

$$\int_0^{h_0}\int_0^{r_0} 10^{-\varepsilon_{BID}\cdot\sqrt{r^2+h^2}\cdot C_{BID}}\,\frac{P}{m_{BID}}\,C_{BID}\,\frac{2\pi r}{4\pi(r^2+h^2)}\,dr\,dh = \Omega\cdot hc/\lambda\cdot L_\Omega$$

where h_0 and r_0 are the height and radius of the BID-produced nanophosphor solution in a 48 well plate, respectively; ε_{BID} is the absorption coefficient of the BID-produced nanophosphor solution at its corresponding peak emission wavelength; C_{BID} is the concentration of the nanophosphor solution; $\Omega = 4\pi$ is the solid angle; hc/λ is the energy of a single photon at the peak emission wavelength, and L_Ω is the radiance measured by the IVIS system.

Then the highest achievable afterglow power density in different organs *in vivo* after intravenous delivery is estimated as follows:

$$I = \int_0^{l_0}\frac{P}{m_{BID}}\,C_{BID,blood}\,v/v_{blood}\,exp(-\mu_{eff}\,l)\,dl$$

where l_0 is the estimated dimension of the mouse organ; $C_{BID,blood}$ is the estimated highest achievable concentration of BID-produced nanophosphor solution in the blood based on previous reports (*52, 53*); v/v_{blood} is the volume percentage of blood in specific organs; μ_{eff} is the effective attenuation coefficient of specific organ tissue at the peak emission wavelength of the nanophosphor based on previous reports (*54*). The results for the blue-emitting nanophosphors are summarized below in table S1:

Table S1. Estimated power densities of systemically delivered nanophosphors in different organs.

	Brain ($\mu W/cm^2$)	Liver ($\mu W/cm^2$)
SMSO	0.627	1.01
SAO	21.8	36.2

Furthermore, we have also compared the estimated highest achievable power density above with the power needed for various biological applications.

First, for optogenetics neuromodulation, it has been previously reported that channelrhodopsins with slow dynamics (such as SSFO, stable step-function opsin) can act as photon integrators, and their photocurrent is determined by the total photon exposure even under extremely low light conditions (*55*, *56*). Specifically, it has been experimentally verified that the apparent time constants for activation of SSFO under ~1.6 and 2.9 $\mu W/cm^2$ 470-nm light are ~59 and 38 s, respectively (*55*). As calculated above, BID-produced SAO nanophosphor colloid can provide enough photons to activate SSFO across the entire brain within 5 s upon intravenous delivery. Furthermore, the recently developed ultra-sensitive step-function opsin (SOUL) requires even less photon exposure at 470-nm than SSFO (*57*), thus enabling SOUL to be activated with a lower SAO dosage or a shorter time interval after delivery.

Second, for light-mediated gene-editing, the single-chain psCas9 based on pdDronpa can be activated with 10 $\mu W/cm^2$ 500-nm light (*13*). The calculations above suggest that the intravenous delivery of SAO can provide enough power density in both the brain and the liver for activating psCas9.

Third, for photodynamic therapy, previous *in vitro* studies have demonstrated that the low fluence threshold for a commercially available photosensitizer $TPPS_{2a}$ under ~0.28 $\mu W/cm^2$ blue light illumination was 12 mJ/cm^2 (*58*). Such power density and light fluence should be achievable within ~5.5 min after intravenous delivery of SAO nanophosphors, while the *in vivo* afterglow intensity can be maintained at a relatively high level by applying constant remote recharging and leveraging the intrinsic circulatory system, as demonstrated in fig. S16. Furthermore, another recent report utilized bioluminescence to locally activate photosensitizer for photodynamic therapy *in vivo*, and the effective bioluminescence radiation was estimated to be as low as 15.6 mJ/cm^2 (26 $\mu W/cm^2$ for 10 min) at 500 nm (*59*). This light fluence requirement can also be achieved by using SAO nanophosphors as an internal light source after intravenous injection with constant remote recharging.

In summary, our calculations above suggest the feasibility of applying the blue-emitting nanophosphor colloids reported in this work as circulation-delivered internal light sources for various advanced biological applications, such as optogenetics neuromodulation based on SSFO or SOUL, light-mediated genome modification based on psCas9, and photodynamic therapy. Compared with conventional methods of light delivery, the systemically delivered internal light source is advantageous as it can reach deep tissue through blood circulation, illuminate the whole organ of interest uniformly, and does not require invasive implants such an LED or an optical fiber. Nonetheless, the advanced biological applications proposed above remain to be explored in future studies.

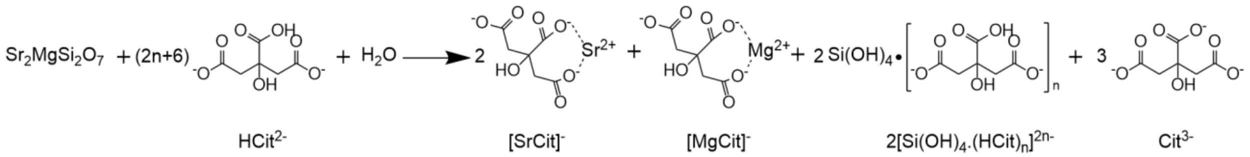

Fig. S1. Hypothesized reaction during the BID process. $HCit^{2-}$ represents the doubly-deprotonated citrate anion, and Cit^{3-} represents the triply-deprotonated citrate anion. Sr^{2+} and Mg^{2+} cations form coordination complexes with the citrate anion with 1:1 stoichiometry (*60*). Triply-deprotonated citrate anions are the dominant form in these complexes in our pH range of interest (e.g., 6~10) (*61*). In addition, it has been reported that silica forms complexes with citrate with unknown stoichiometry (thus the subscript of *n*) to dissolve in an aqueous solution (*62*).

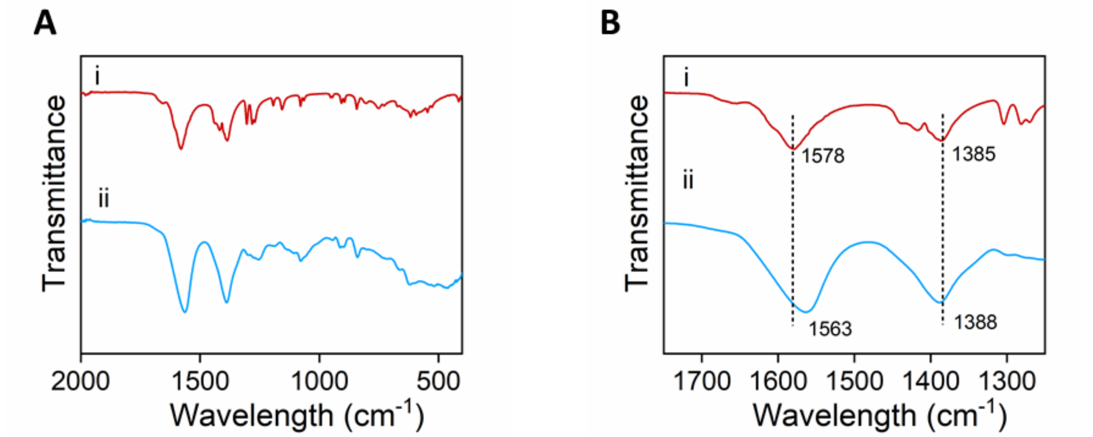

Fig. S2. FTIR spectra of trisodium citrate and the BID solution. Full-range (**A**) and close-up view (**B**) of FTIR spectra of trisodium citrate (i) and the citrate etchant after dissolving SMSO bulk particles (ii), revealing the chemical identity of coordinated metal cations in the solution after dissolution. The significant downshift of the antisymmetric stretching band of —COO^{-} [i.e., υ_{as}(—COO^{-})] from 1578 cm^{-1} to 1563 cm^{-1} indicates the formation of the metal-carboxylate bond (*63*, *64*). Therefore, coordinated metal cations such as —COOMg, —COOSr, —COOEu, and —COODy should exist in the solution after dissolution. In addition, the upshift of the symmetric stretching band of —COO^{-} [i.e., υ_{s}(—COO^{-})] from 1385 cm^{-1} to 1388 cm^{-1} is attributed to the formation of the metal-carboxylate bond. Based on this data, we postulate that metal-citrate complex ions are the most probable form of dissolved metals after dissolution of bulk SMSO.

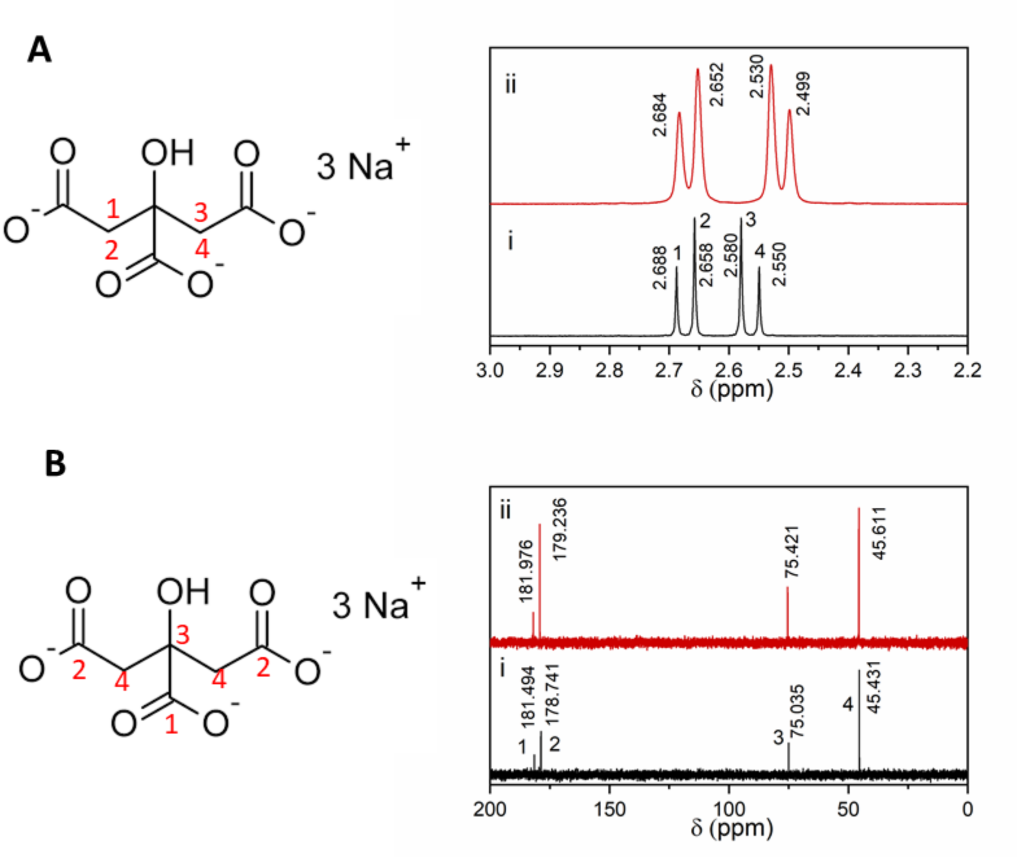

Fig. S3. NMR spectra of trisodium citrate and the BID solution. ^{1}H (**A**) and ^{13}C (**B**) NMR spectra of trisodium citrate (i) and the citrate etchant after dissolving SMSO bulk particles (ii), confirming the formation of metal-carboxyl bonds in the solution. Previous work reported a downshift of α-H atoms with peak broadening and an upshift of all C atoms when citrate forms coordination complexes with metal cations (*65*, *66*).

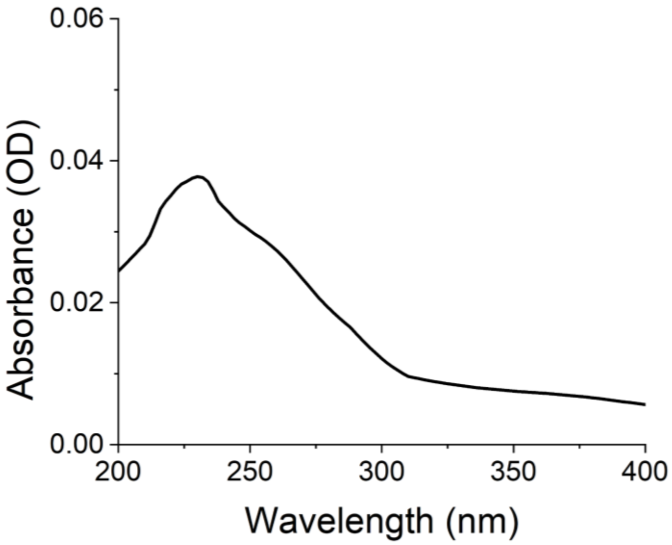

Fig. S4. UV absorption spectrum of the BID solution. UV absorption spectrum of the citrate etchant after dissolving SMSO bulk phosphor reveals the formation of silicate-citrate complexes. Such complexes were implied by a previous work studying the dissolution of silica by citrate (*67*), while an absorption peak near 240 nm suggests the formation of silicate-citrate complexes due to charge transfer (*62*).

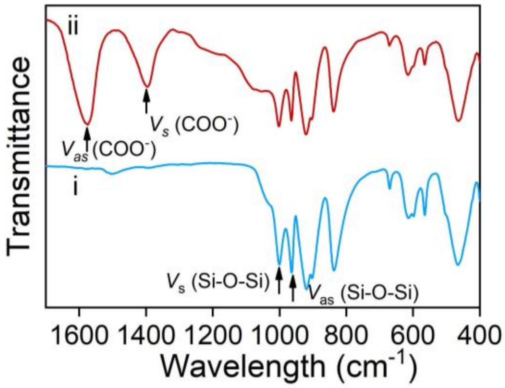

Fig. S5. FTIR spectra of SMSO bulk phosphor (i) and colloidal nanophosphor (ii). Peaks at 964 cm^{-1} and 1000 cm^{-1} correspond to Si-O-Si stretching modes (*68*, *69*), which are missing from the IR spectrum of the etched solution (fig. S2). This result suggests the absence of Si-O-Si bonds in the soluble products after dissolution, thus confirming a soluble form of silica-citrate complex in fig. S1. Additionally, the presence of symmetric and antisymmetric stretching bands of $—COO^-$ [i.e., $\upsilon_s(—COO^-)$ and $\upsilon_{as}(—COO^-)$] as labeled in ii suggests the adsorption of citrate anions on the surface of SMSO colloid to improve its colloidal stability when dispersed in water.

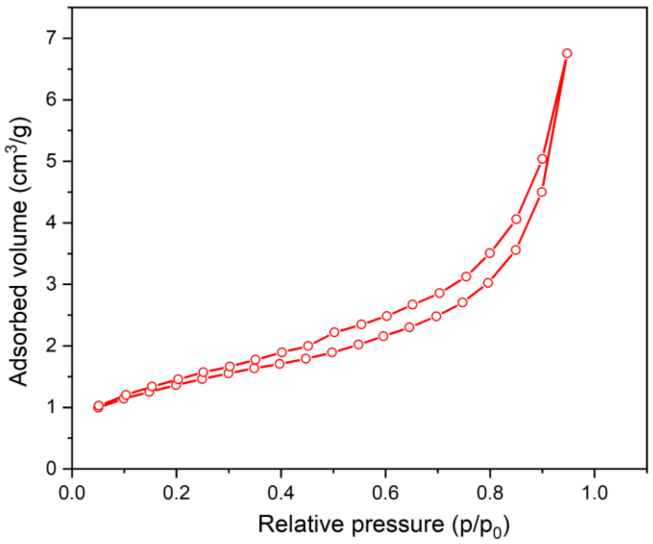

Fig. S6. BET isotherm of SMSO bulk particles prior to the BID process. We obtained a specific surface area of 43,850 cm^2/g from this isotherm, thus yielding a total surface area of 4,385 cm^2 for kinetically preserved dissolution in the CC experiments.

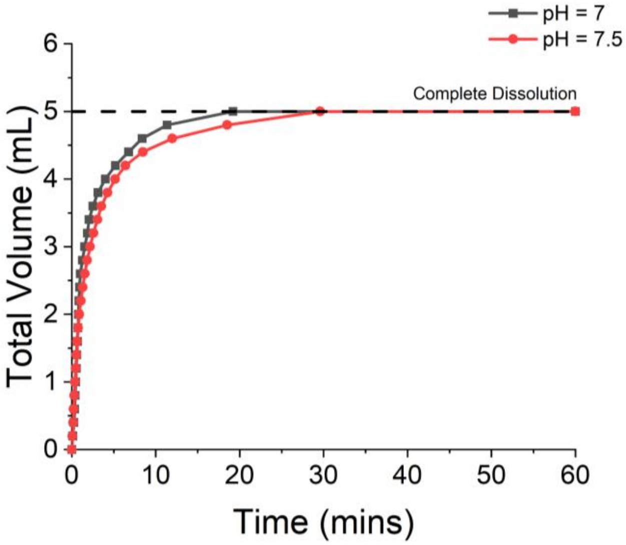

Fig. S7. Titration curves at lower pH. Plots of titrant volume as a function of time for complete dissolution of SMSO at pH 7 and pH 7.5 with the CC technique (see Materials and Methods).

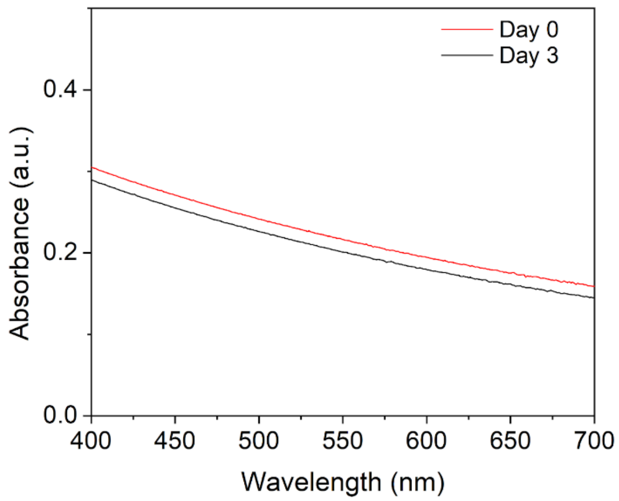

Fig. S8. UV absorption spectra of SMSO colloidal solutions. UV spectra of two solutions corresponding to Fig. 1J, bottom row, reveal the resistance of critically sized nanoparticles to further dissolution. Note that the 400-nm absorbance of SMSO colloidal solution in water is proportional to its molar concentration by an extinction coefficient of 2.7×10^8 L/mol/cm.

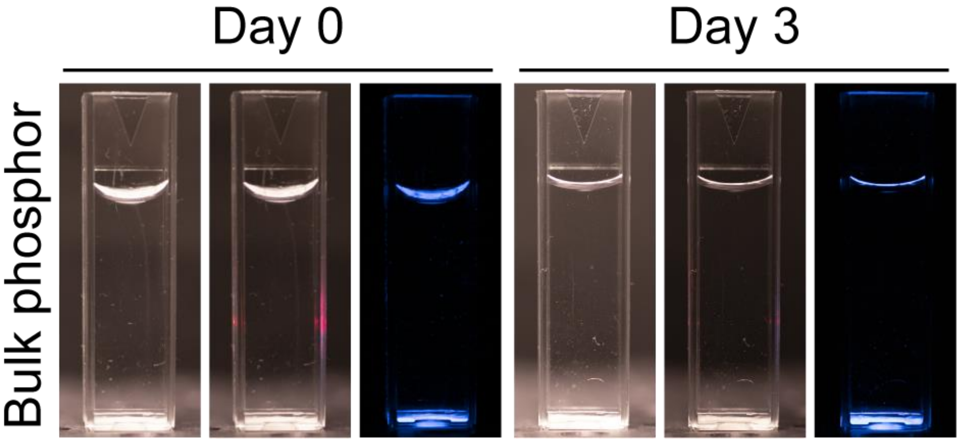

Fig. S9. Images of bulk SMSO particles in pure water. Bright-field image (left), Tyndall effect (middle), and afterglow image (right) of bulk SMSO particles in pure water without citrate (pH = 7) before and after mixing for 3 days under the same experimental conditions as the BID process with citrate, showing negligible dissolution of SMSO in pure water. This result ruled out the possibility of SMSO dissolving in pure water, which is consistent with the limited aqueous solubility of alkaline earth silicates. The lack of dissolution in pure water strongly contrasts with complete dissolution in citrate buffer at the same pH (fig. S7). Taken together, these results confirm the hypothesis that citrate ions provide the main drive for a negative $\triangle g$, while the pH modulates undersaturation due to the consumption of protons in the reaction (fig. S1).

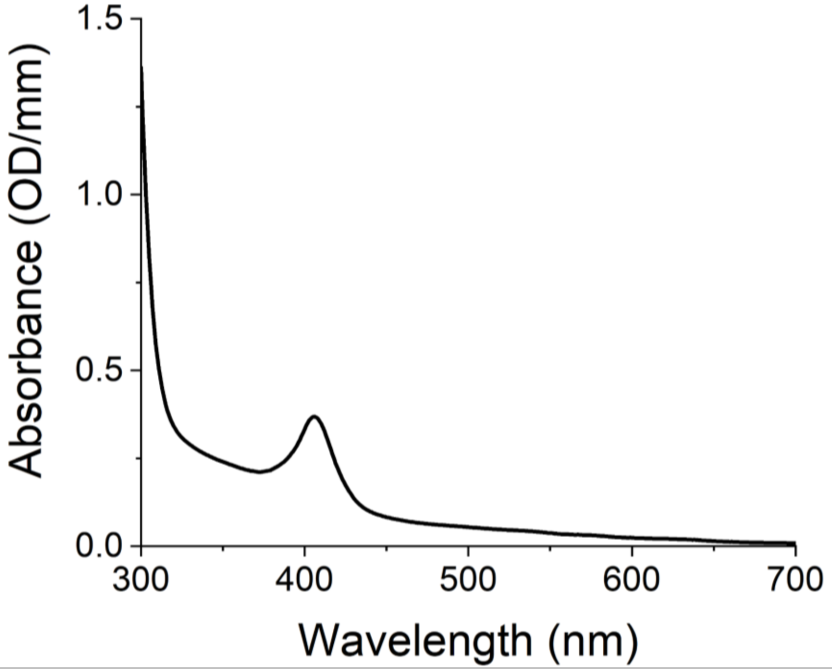

Fig. S10. The absorption spectrum of FBS in the 300-700 nm range with a 1 mm optical path. Note the absorbance of 0.06 mm^{-1} at 470 nm, the emission wavelength of the SMSO colloid. This absorbance value corresponds to the attenuation of 56% in a microcentrifuge tube, agreeing with ~50% reduction in the afterglow of SMSO colloid in FBS observed in Fig. 2F.

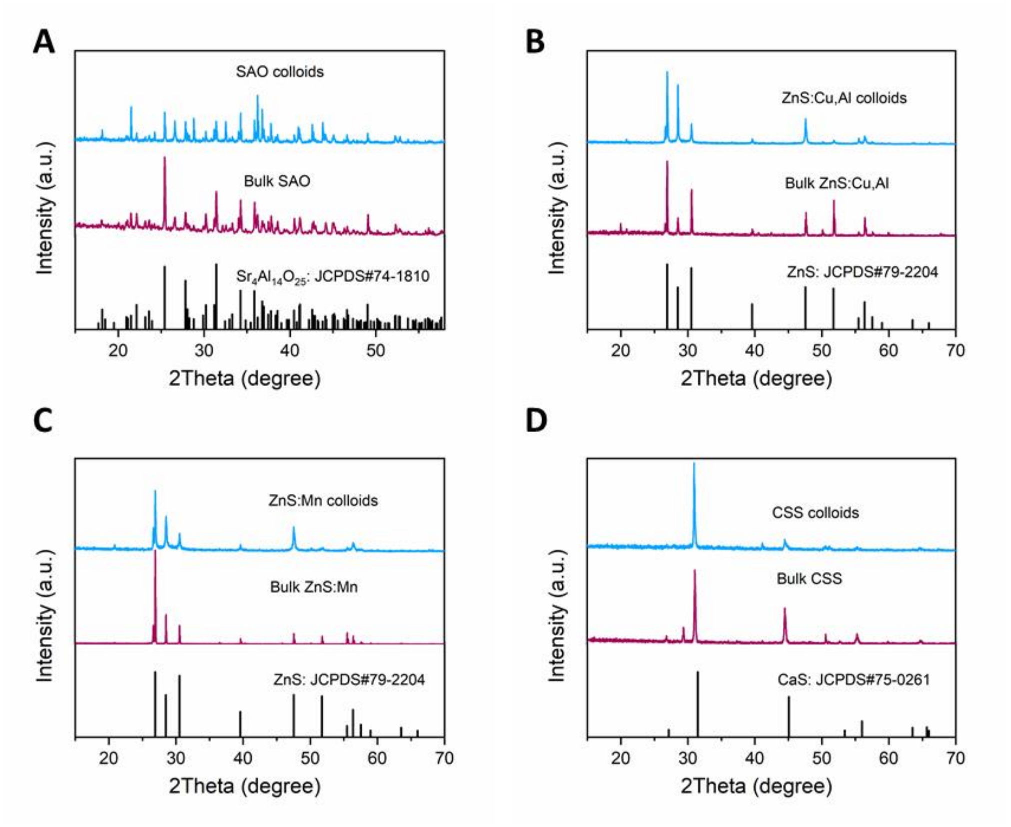

Fig. S11. XRD spectra of different phosphors. XRD spectra of bulk phosphors and their corresponding colloids for different materials: (**A**) SAO; (**B**) ZnS:Cu,Al; (**C**) ZnS:Mn; and (**D**) CSS.

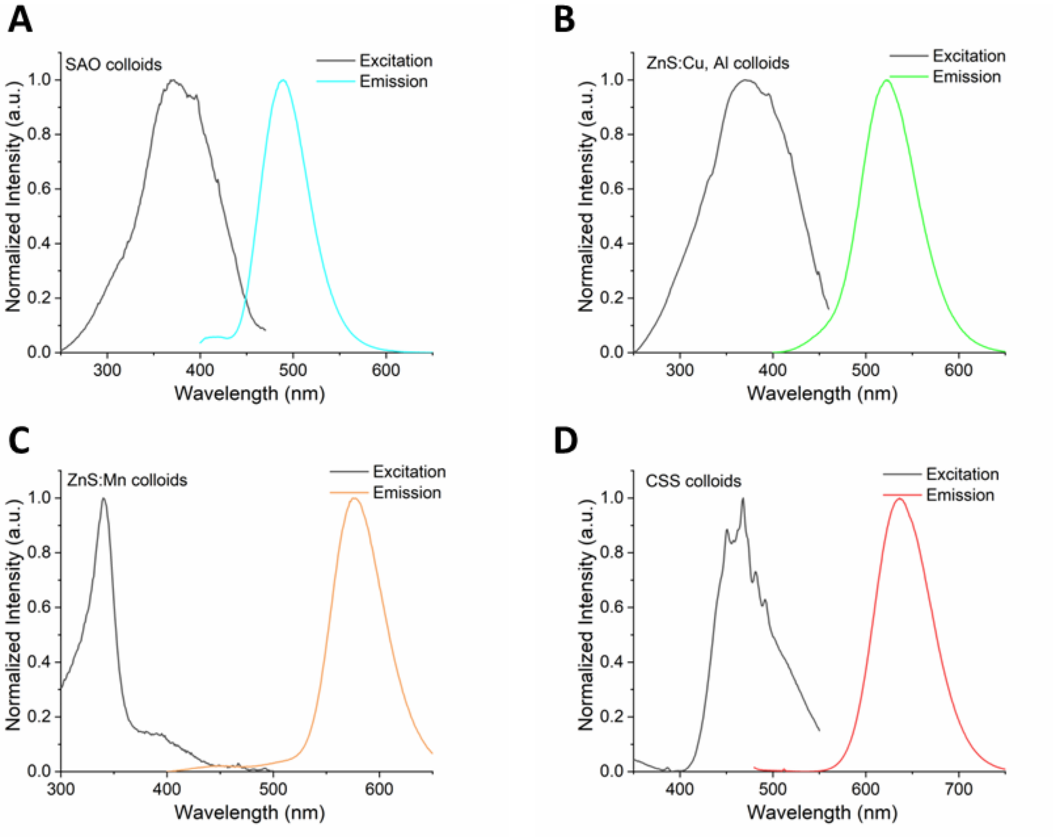

Fig. S12. Photoluminescence characterization of different colloidal nanophosphors. The excitation and emission spectra of different nanophosphor colloids produced by the BID approach: (**A**) SAO; (**B**) ZnS:Cu,Al; (**C**) ZnS:Mn; and (**D**) CSS.

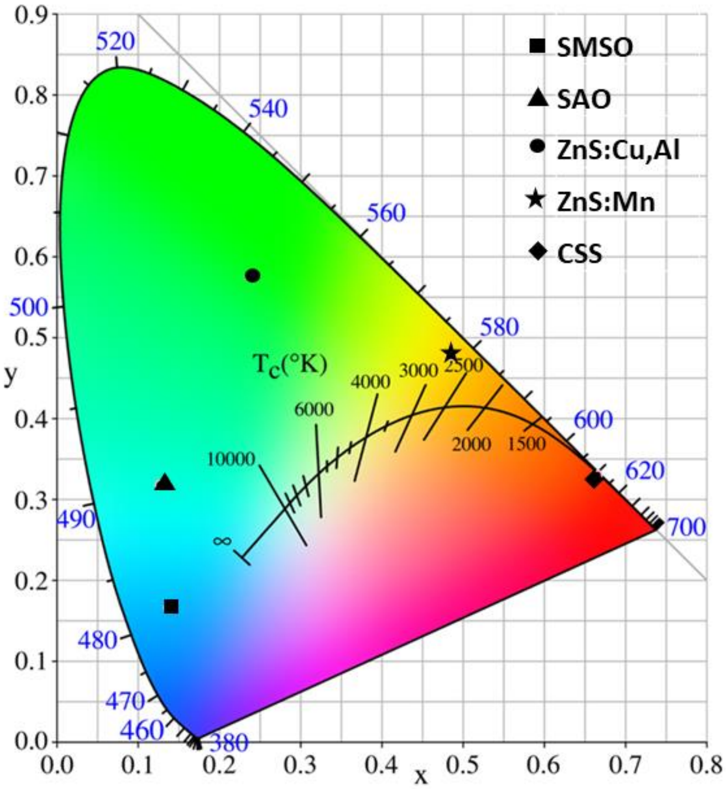

Fig. S13. CIE chromaticity diagram of five BID-produced nanophosphor colloids.

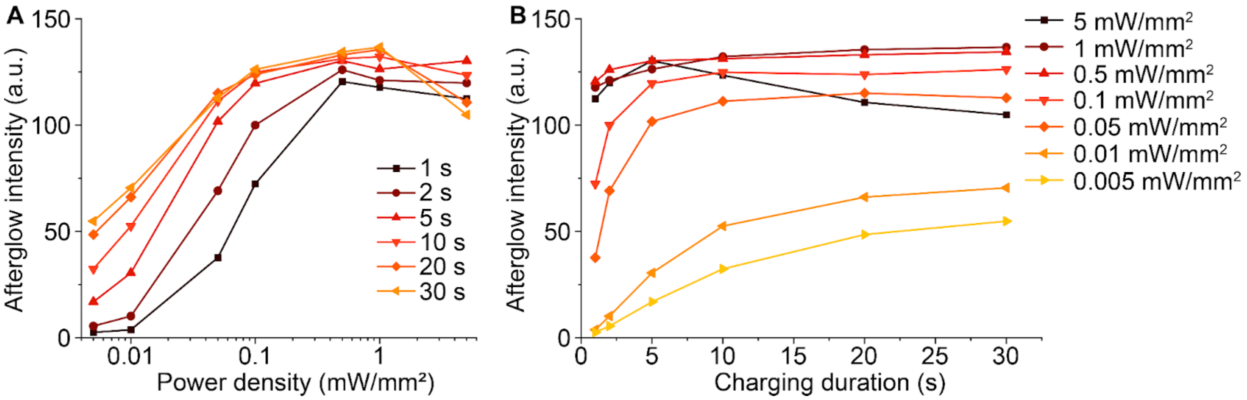

Fig. S14. Optimization of the afterglow intensity of the SMSO colloid by varying the power density and duration of recharging. The afterglow intensity is plotted against the recharging power density and duration in (**A**) and (**B**), respectively.

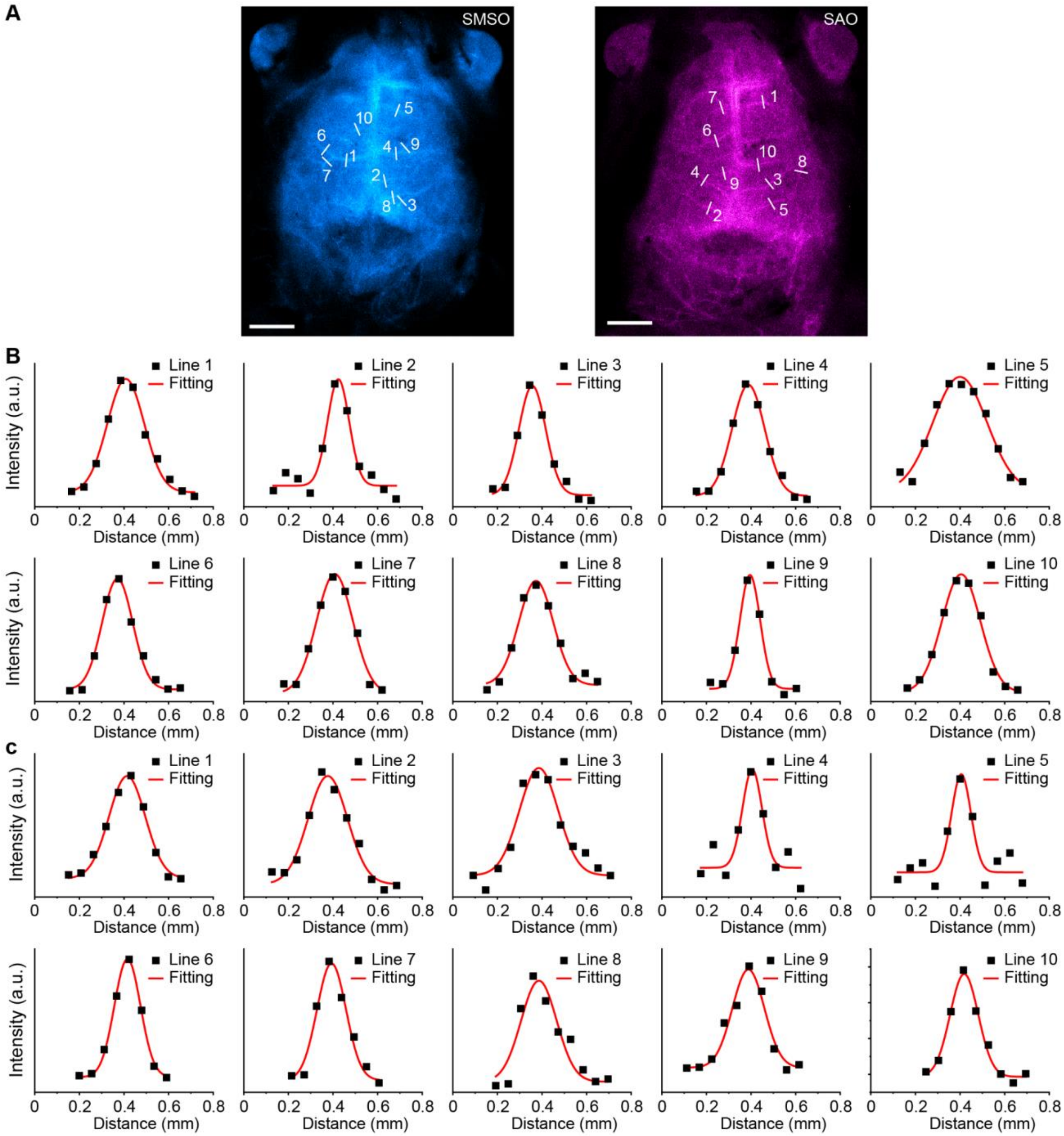

Fig. S15. Vessel width analysis of transcranial afterglow brain imaging. (**A**) Transcranial afterglow images of brain vessels after intravenous injection of SMSO (left) and SAO (right) colloidal solutions. Vessels used for width analysis are highlighted with short white lines. Scale bars represent 2.5 mm. (**B,C**) Raw and fitted line profiles for the SMSO (**B**) and SAO (**C**) afterglow images in (**A**).

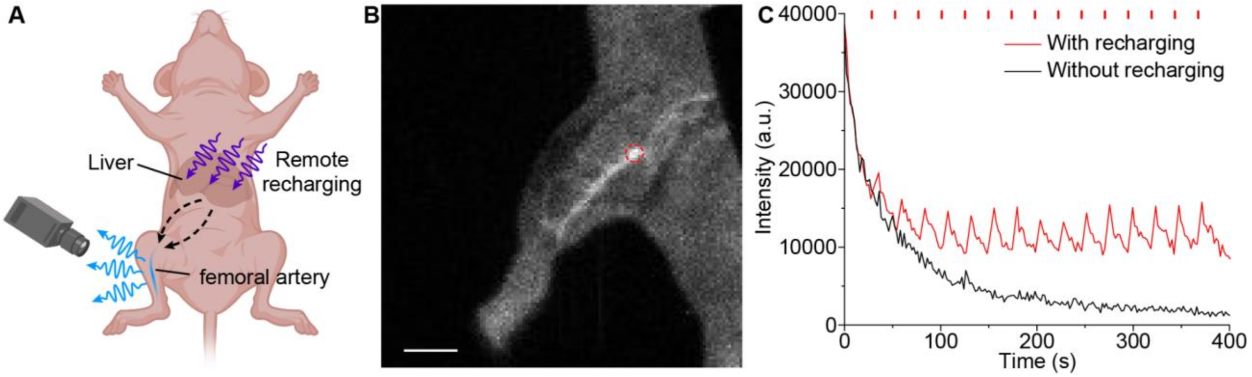

Fig. S16. Remote recharging of circulating nanophosphor colloids *in vivo*. (**A**) Schematic showing remote recharging of circulating nanophosphor colloids in hepatic vessels and afterglow imaging of the femoral artery in the mouse hindlimb. The intrinsic circulatory system of the mouse brings the charged nanophosphors in the hepatic vessels to the femoral artery. (**B**) An afterglow image of the femoral artery. The dashed red circle highlights the region for analyzing the afterglow intensity dynamics. The scale bar represents 5 mm. (**C**) Afterglow intensity dynamics of the femoral artery with (red) or without (black) remote recharging in hepatic vessels. The red ticks above the plot represent periodic remote recharging events.

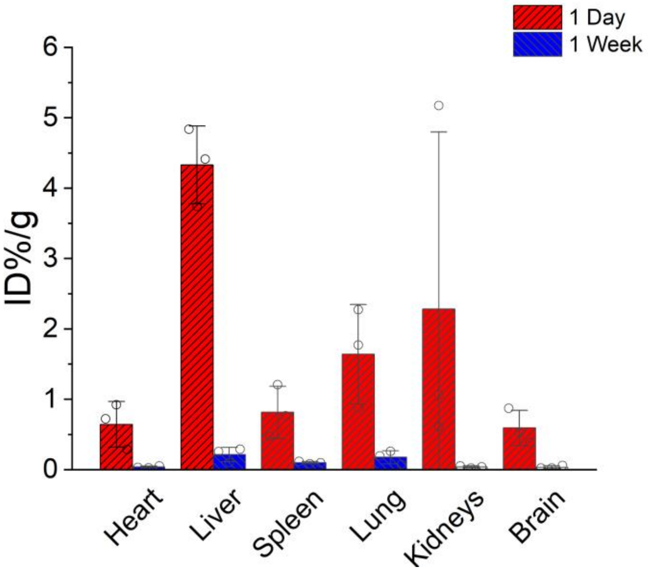

Fig. S17. Biodistribution of systemically delivered SMSO colloids (493 nmol/L) in major organs on one day (red bars) and one week (blue bars) post-injection. All data are presented as mean values +/- SD, with individual data shown in open circles. *n* = 3 mice for both time points.

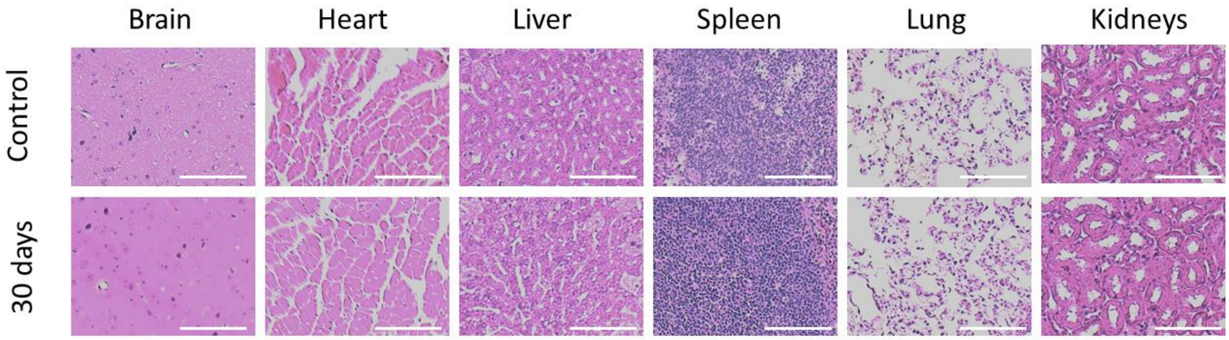

Fig. S18. Histological analysis of mouse organs. H&E stained images of major mouse organs 30 days after intravenous injection of an SMSO colloidal solution (493 nmol/L, bottom row), in comparison with those from the control group (top row). No noticeable tissue damage or pathological lesion was found in organs of colloid-injected mice. Scale bars represent 100 μm.

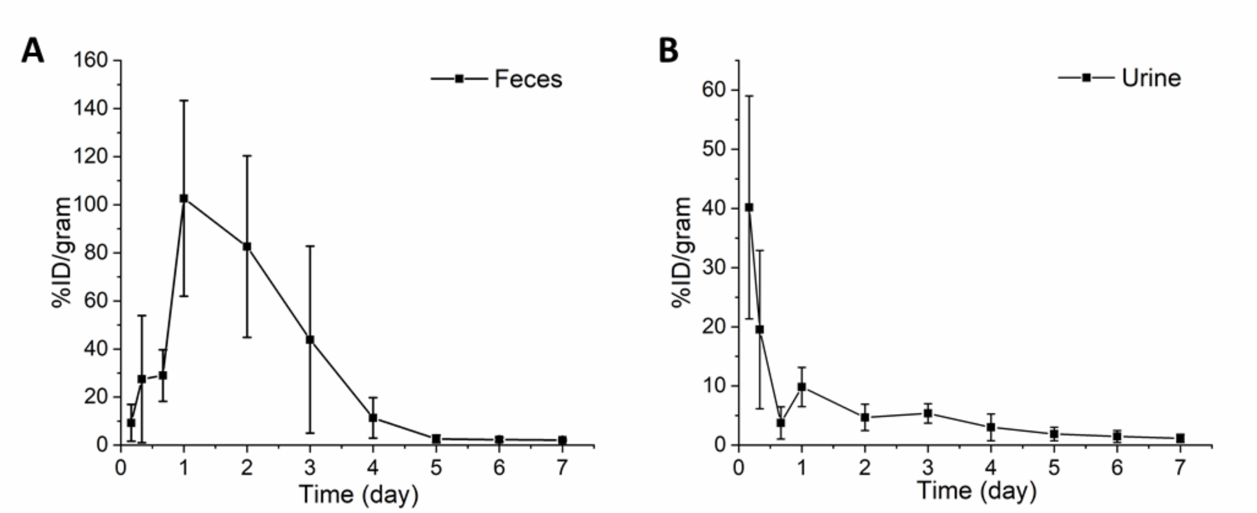

Fig. S19. Clearance study of SMSO nanophosphor colloids *in vivo*. The excretion profile of intravenously injected SMSO colloids in the feces (**A**) and urine (**B**) All data are presented as mean values +/- SD. (*n* = 3 mice).

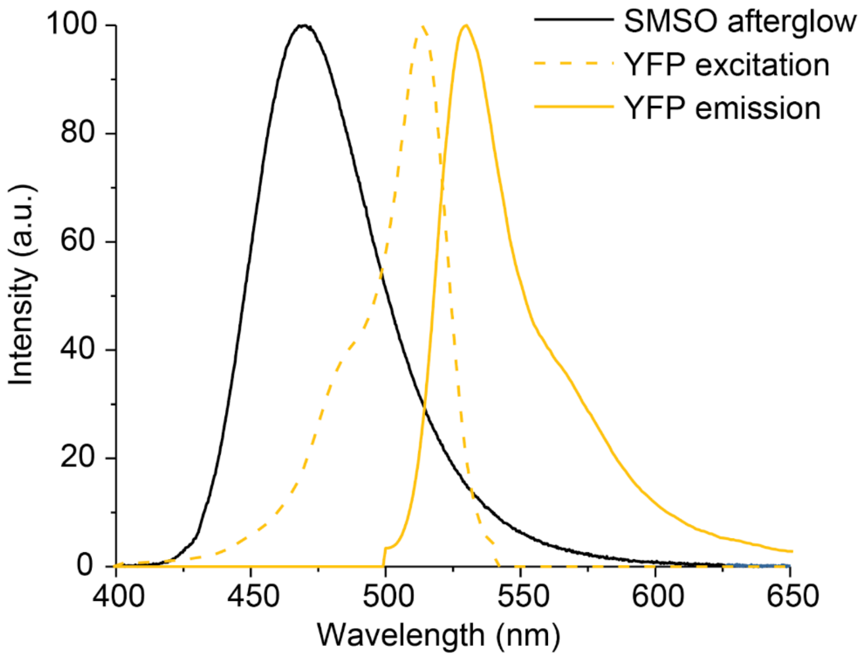

Fig. S20. The afterglow spectrum of SMSO colloid overlaid with the excitation and emission spectra of the YFP.

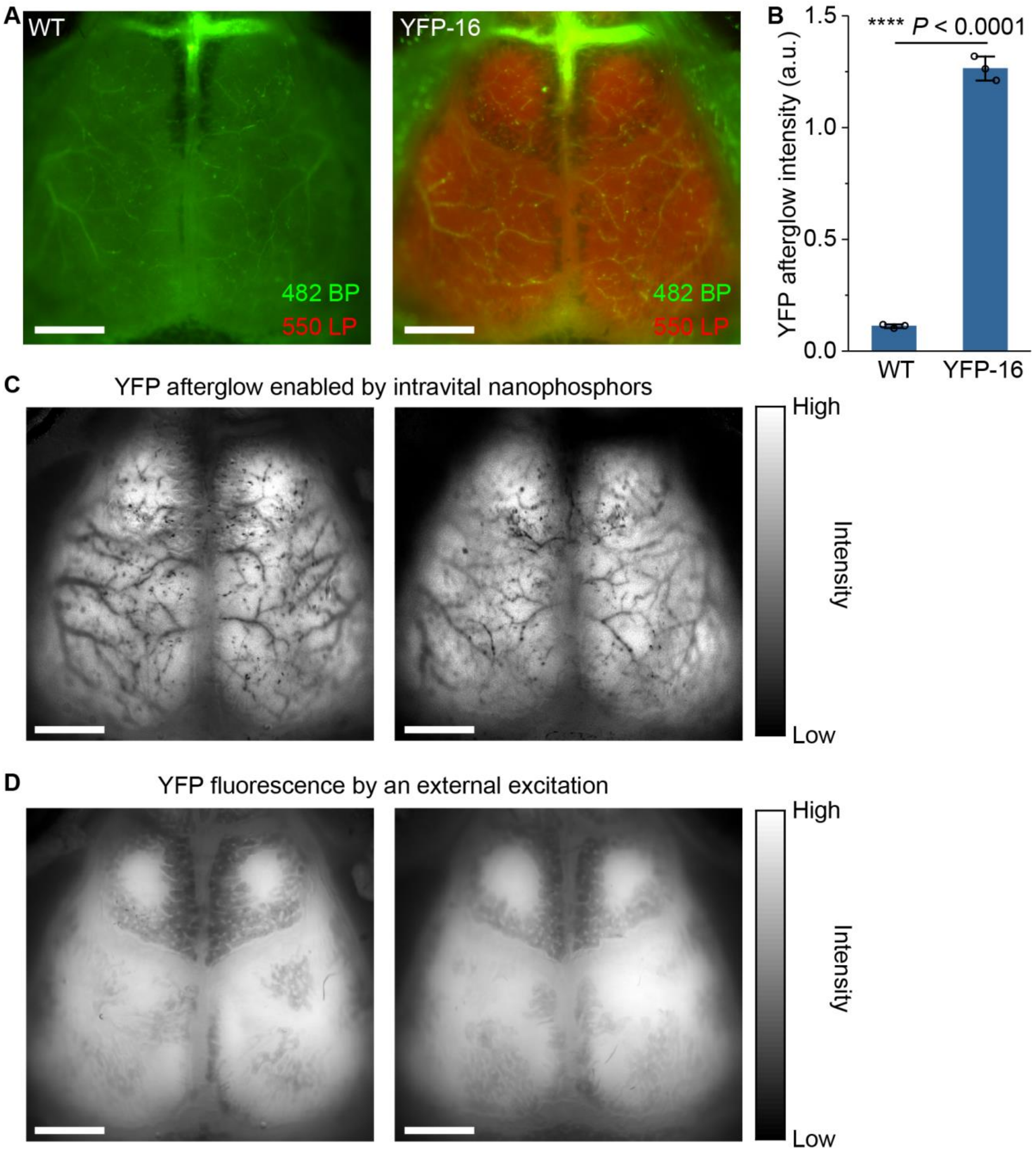

Fig. S21. YFP imaging with intravital and external excitation sources. (**A**) Overlay of raw afterglow images collected with 482BP (green, collecting SMSO afterglow only) or 550LP (red, collecting YFP afterglow only) filters in the brains of WT (left) and YFP-16 (right) mice in Fig. 5D. (**B**) Statistical analysis of YFP afterglow intensity of WT and YFP-16 mice in Fig. 5D. All data are presented as mean values +/- SD. $n = 3$ for all groups. (One-way ANOVA, $F(1,4) = 1344.12$, $P < 0.0001$). (**** $P < 0.0001$) (**C,D**) Additional YFP afterglow images via an intravital light source (**C**) and YFP fluorescence images via an external excitation (**D**) in the brains of two additional YFP-16 mice. All scale bars represent 2 mm.

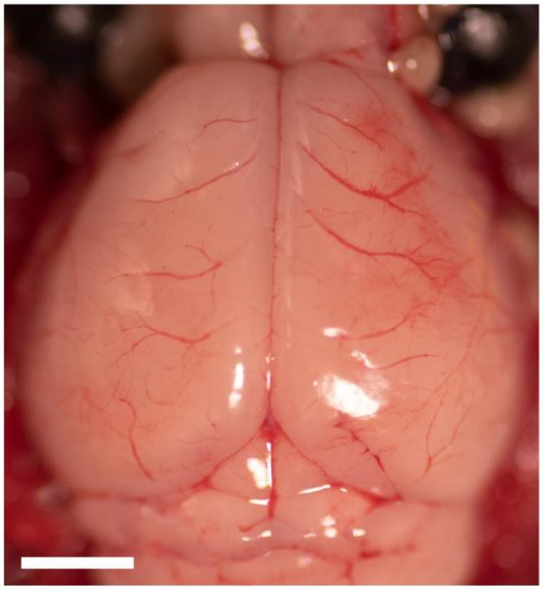

Fig. S22. Photo of the mouse brain shown in Fig. 5B after removing the skull. The scale bar represents 2 mm.

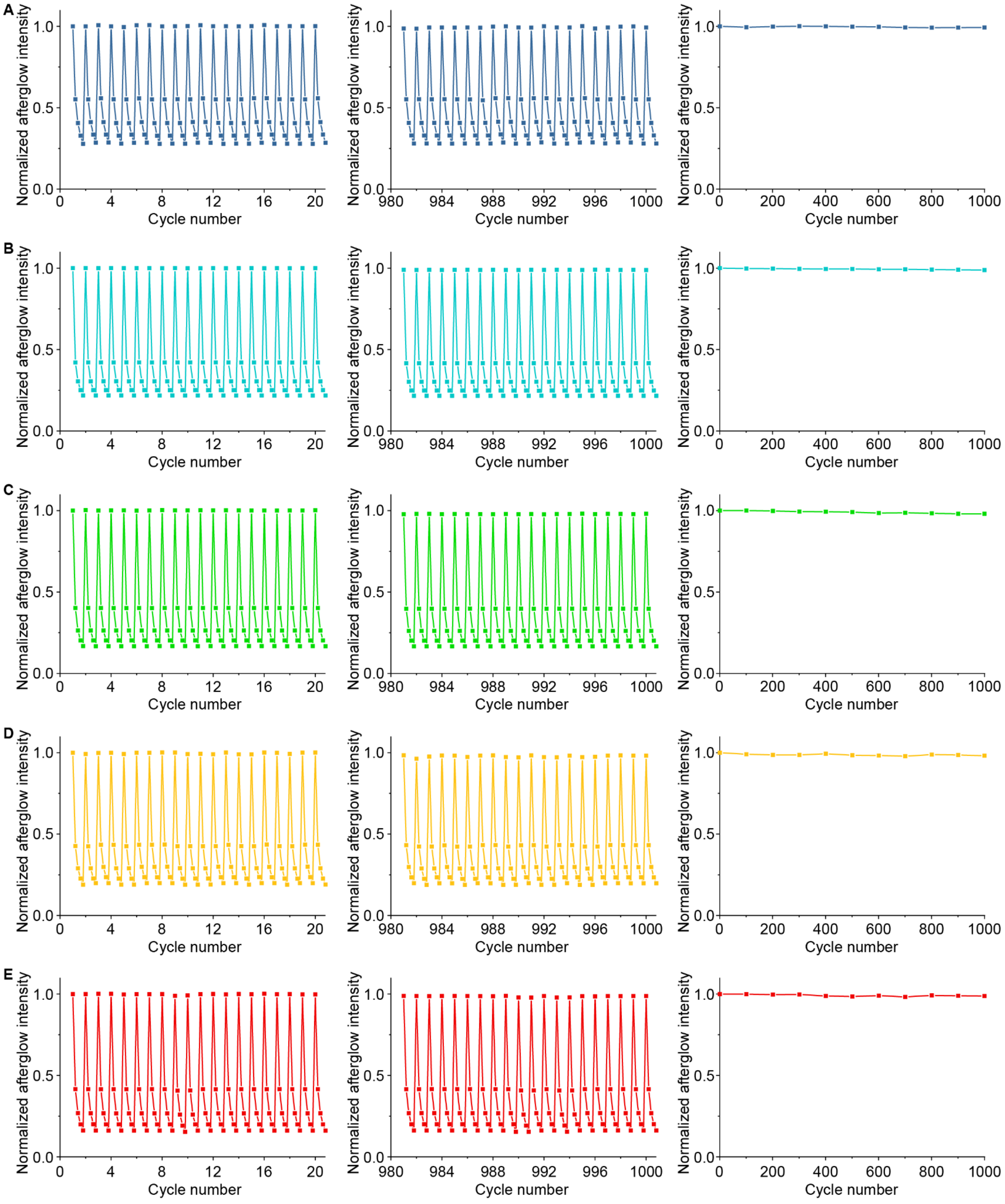

Fig. S23. Photostability of nanophosphor colloids. The afterglow intensity of SMSO (**A**), SAO (**B**), ZnS:Cu,Al (**C**), ZnS:Mn (**D**), and CSS (**E**) in the first 20 (left), last 20 (middle), and every 100 (right) recharging cycles.

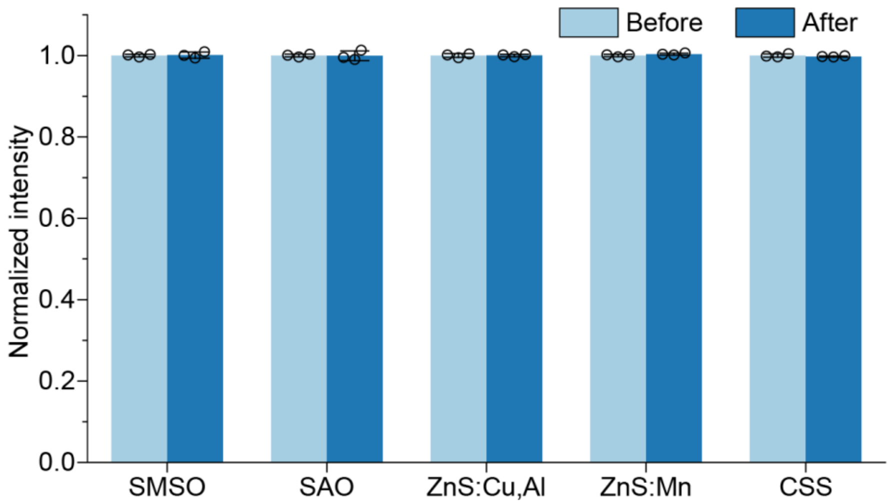

Fig. S24. Normalized intensity of SMSO, SAO, ZnS:Cu,Al, ZnS:Mn, and CSS before and after 24-hr continuous bleaching by 1 mW/mm^2 365-nm light. The data are represented as mean ± SD. *n* = 3 for all groups.

Table S2. Comparison of BID-produced nanophosphors with previous reports.

	Materials	Emission Wavelength	Half-life Time	*In vivo* afterglow intensity of subcutaneous injection	*Ex vivo* afterglow intensity	Sizes	References
**Inorganic Persistent Phosphors**							
1	$Sr_2MgSi_2O_7$:Eu,Dy	470 nm	60 s	1.9×10^9 p/s/cm^2/sr/(mg/mL)	2.0×10^{10} p/s/cm^2/sr/(mg/mL)	43±12nm	This study
	$Sr_4Al_{14}O_{25}$:Eu,Dy	490 nm	77 s	3.8×10^{10} p/s/cm^2/sr/(mg/mL)	6.2×10^{11} p/s/cm^2/sr/(mg/mL)	69±19 nm	
	ZnS:Cu,Al	534 nm	52 s	7.5×10^8 p/s/cm^2/sr/(mg/mL)	3.6×10^9 p/s/cm^2/sr/(mg/mL)	56±9 nm	
	ZnS:Mn	578 nm	56 s	6.8×10^8 p/s/cm^2/sr/(mg/mL)	3.2×10^9 p/s/cm^2/sr/(mg/mL)	88±22 nm	
	$Ca_{0.85}Sr_{0.15}S$:Eu,Tm	650 nm	52 s	3.0×10^8 p/s/cm^2/sr/(mg/mL)	1.4×10^9 p/s/cm^2/sr/(mg/mL)	86±26 nm	
2	$SiO_2/CdSiO_3$: In^{3+}	438 nm	~25 s	~1 x 10^7 (p/s/cm^2/sr)/(mg/mL)	Absolute intensity not reported for *ex vivo* imaging	~70 nm	(70)
	$SiO_2/CdSiO_3$: In^{3+},Mn^{2+}	580 nm	~35 s	~5 x 10^7 (p/s/cm^2/sr)/(mg/mL)			
	$SiO_2/CdSiO_3$: In^{3+},Tb^{3+}	549 nm	~35 s	~4 x 10^7 (p/s/cm^2/sr)/(mg/mL)			

	$SiO_2/CdSiO_3$: In^{3+},Dy^{3+}	578 nm	~45 s	Subcutaneous injection not performed			
3	$ZnGa_2O_4$:$Cr_{0.004}$	696 nm	~60 s	~5 x 10^6 (p/s/cm^2/sr) /(mg/mL)	~4 x 10^7 (p/s/cm^2/sr) /(mg/mL)	~9 nm	(17)
4	$Ca_{0.2}Zn_{0.9}Mg_{0.9}Si_2O_6$: Eu^{2+}, Dy^{3+}, Mn^{2+}	690 nm	~60 s	~5 x 10^5 (p/s/cm^2/sr) /(mg/mL)	*Ex vivo* imaging not performed	50-100 nm	(18)
5	$ZnGa_2O_4$:Cr^{3+}-BSA-TAMRA	702 nm	~2 s	~6 x 10^6 (p/s/cm^2/sr) /(mg/mL)	*Ex vivo* imaging not performed	59 nm	(10)
6	$ZnGa_{1.995}Cr_{0.005}O_4$	695 nm	~200 s (excited with 5700 lumen LED array); ~1000 s (exited with 6W UV lamp)	Subcutaneous injection not performed	*Ex vivo* imaging not performed	80 nm	(8)
7	Si-Pc functionalized $Zn_{1.25}Ga_{1.5}Ge_{0.25}O_4$:0.5%$Cr^{3+}$,2.5%$Yb^{3+}$, 0.25%$Er^{3+}$ Core@hSiO_2@CCM	695 nm	~15 s	Subcutaneous injection not performed	Absolute intensity not reported for *ex vivo* imaging	176 nm	(71)

8	$ZnGa_2O_4:Cr^{3+}/Sn^{4+}$@MSNs-PEG	695 nm	~15 s	Absolute intensity not reported for subcutaneous afterglow imaging.	Absolute intensity not reported for *ex vivo* imaging.	103 nm	(72)
9	HA-Gd_2O_3-$ZnGa_2O_4:Cr^{3+}$	695 nm	~10 s	Subcutaneous injection not performed.	Absolute intensity not reported for *ex vivo* imaging.	142 nm	(73)
10	c(RGDyK)-PEG-$Zn_{2.94}Ga_{1.96}Ge_2O_{10}:Cr^{3+},Pr^{3+}$	700 nm	~seconds	Absolute intensity not reported for subcutaneous afterglow imaging.	Absolute intensity not reported for *ex vivo* imaging	132 nm	(74)
11	Raspberry-like mesoporous $Zn_{1.07}Ga_{2.34}Si_{0.98}O_{6.56}:Cr_{0.01}$	698 nm	~100 s	Subcutaneous injection not performed	Absolute intensity not reported for *ex vivo* imaging	~110 nm	(75)

12	Folate acid-functionalized $Zn_{1.25}Ga_{1.5}Ge_{0.25}O_4$:0.5%$Cr^{3+}$,2.5%$Yb^{3+}$, 0.25%$Er^{3+}$	691 nm	~350 s	Absolute intensity not reported for subcutaneous afterglow imaging	*Ex vivo* imaging not performed	44 nm	(76)
13	$mSiO_2$@$Gd_3Ga_5O_{12}$:Cr^{3+}, Nd^{3+}	745 nm	~30 s	Absolute intensity not reported for subcutaneous afterglow imaging	Absolute intensity not reported for *ex vivo* imaging	50 nm	(77)
14	$Zn_{1+x}Ga_{2-2x}Ge_xO_4$:Cr^{3+}@zeolitic imidazolate framework-8	699 nm	~40 s	Subcutaneous injection not performed	Absolute intensity not reported for *ex vivo* imaging	100-200 nm	(78)
15	$LaAlO_3$:Cr^{3+},Sm^{3+}	735 nm and 750 nm	~60 s	Subcutaneous injection not performed	*Ex vivo* intensity measurement not performed on the suspension of nanoparticles	<100 nm	(79)
16	$ZnGa_2O_4Cr_{0.004}$	696 nm	~90 s	Subcutaneous injection not performed	Absolute intensity not reported for *ex vivo*	10 nm	(80)

					imaging		
17	$CaMgSi_2O_6:Eu^{2+},Mn^{2+}, Pr^{3+}$	685 nm	~270 s	Subcutaneous injection not performed	*Ex vivo* imaging not performed		(81)
18	Core–shell-structured Ln-doped $NaY(Gd)F_4$ (Ln = Nd^{3+}, Ho^{3+}, Tm^{3+}, Er^{3+})	1064 nm (Nd^{3+})	~250 s	Subcutaneous injection not performed	Absolute intensity not reported for *ex vivo* imaging	42 nm	(16)
		1180 nm (Ho^{3+})	~200 s				
		1475 nm (Tm^{3+})	~100 s				
		1525 nm (Er^{3+})	~100 s				
19	$ZnGa_2O_4:Cr$	700 nm	~180 s	Absolute intensity not reported for subcutaneous afterglow imaging	Absolute intensity not reported for *ex vivo* imaging	141 nm	(82)
20	$(Zn_2SiO_4:Mn):Y^{3+}, Yb^{3+}, Tm^{3+}$	525 nm	~420 s	Subcutaneous injection not performed	Concentration of nanoparticles not reported for *ex vivo* imaging	120-160 nm	(83)

21	$CaSnO_3:Bi^{2+}$	810 nm	~970 s	Subcutaneous injection not performed	Absolute intensity not reported for *ex vivo* imaging	50-100 nm	(84)
22	$LiGa_5O_8:Cr^{3+}$	716 nm	~2900 s	~2 x 10^6 (p/s/cm^2/sr) /(mg/mL)	~2 x 10^6 (p/s/cm^2/sr) /(mg/mL)	50-150 nm	(19)
23	$Zn_{1.1}Ga_{1.8}Ge_{0.1}O_4$:0.5% Cr core and β-$NaYbF_4$:Tm@$NaYF_4$ shell nanocluster	700 nm	~5 s	Subcutaneous injection not performed	Absolute intensity not reported for *ex vivo* imaging	~100 nm	(39)
**Organic Persistent Phosphors**							
24	SPPVN	780 nm	288 s	~1.0 x 10^7 (p/s/cm^2/sr) /(mg/mL)	~1.2 x 10^8 (p/s/cm^2/sr) /(mg/mL)	24 nm	(85)
25	MEHPPV-based SPN-NCBS	780 nm	396 s	~5.7 x 10^8 (p/s/cm^2/sr) /(mg/mL)	~1.8 x 10^9 (p/s/cm^2/sr) /(mg/mL)	40 nm	(9)
26	DPhCzT-based OSN-T / OSN-B	530 nm	<1 s	~1.3 x 10^7 (p/s/cm^2/sr) /mM	~1.3 x 10^7 (p/s/cm^2/sr) /mM	20-60 nm	(86)
27	PFVA-NCBS-DO	780 nm	~180 s	~1.8 x 10^8 (p/s/cm^2/sr) /(mg/mL)	~1.1 x 10^{10} (p/s/cm^2/sr) /(mg/mL)	~125 nm	(11)
28	Sodium alginate gel loaded with PdPc(OBu)	613 nm	~1 s	~2.2 x 10^2 (p/s/cm^2/sr) /(mg/mL)	~2.2 x 10^2 (p/s/cm^2/sr) /(mg/mL)	~100 nm	(87)

	$_8$-4-(5,6-dihydro-2-phenyl-1,4-oxathiin-3-yl)-N,N-dimethylbenzenamine-Eu(TPPO)$_2$(β-NTA)-based nanoparticles						
29	Si-Pc-CUEM afterglow nanoparticles	613 nm	~1.5 s	Absolute intensity not reported for subcutaneous afterglow imaging	Absolute intensity not reported for *ex vivo* imaging	~225 nm	(14)
30	UCANPs@RAW	615 nm	~17 s	Absolute intensity not reported for subcutaneous afterglow imaging	Absolute intensity not reported for *ex vivo* imaging	~320 nm	(88)
31	AGL AIE dots	650 nm	~2880 s	Subcutaneous injection not performed	~1.5 x 10^8 (p/s/cm^2/sr) /(mg/mL)	~95 nm	(89)

Abbreviations: PEG: polyethylene glycol; BSA: bovine serum albumin; TAMRA: 5-carboxy-tetramethylrhodamine; Si-Pc: silicon phthalocyanine; $hSiO_2$: hollow silica; CCM: cancer cell membrane; MSNs: mesoporous silica nanoparticles; HA: hyaluronic acid; c(RGDyK): cyclic arginine-glycine-aspartic acid-tyrosine-lysine pentapeptide; $mSiO_2$: mesoporous silica; PPV: amphiphilic poly(*p*-phenylenevinylene); SPPVN: self-assembled amphiphilic poly(*p*-phenylenevinylene) nanoagents; MEHPPV: poly[2-methoxy-5-(2-ethylhexyloxy)-1,4-phenylene] end-capped with dimethylphenyl ; SPN:semiconducting polymer nanoparticles; NCBS: silicon 2,3-naphthalocyanine bis(trihexylsilyloxide); DPhCzT: 4,6-Diphenyl-2-carbazolyl-1,3,5-triazine; OSN-T/B: organic semiconducting nanoparticles synthesized via Top-down/Bottom-up approach;

PFVA: poly[(9,9′-dioctyl-2,7-divinylenefluorenylene)-alt-(9,10-anthracene)]; DO: (N,N-dimethyl-4-(3-phenyl-5,6-dihydro-1,4-dioxin-2-yl)aniline); $PdPc(OBu)_8$: 1,4,8,11,15,18,22,25-octabutoxyphthalocyaninato-palladium(II); TPPO: Triphenylphosphine oxide: NTA: nitrilotriacetic acid; UCANPs@RAW: $PdPc(OBu)_8$, 4-(5,6-dihydro-2-phenyl-1,4-oxathiin-3-yl)-N,N-dimethylbenzenamine, and $Eu(TTA)_3Phen$-based macrophage-camouflaged afterglow nanocomplex; AGL: afterglow luminescent; AIE: aggregation-induced emission.

REFERENCES AND NOTES

1. G. Hong, A. L. Antaris, H. Dai, Near-infrared fluorophores for biomedical imaging. *Nat. Biomed. Eng.* **1**, 0010 (2017).

2. L. Fenno, O. Yizhar, K. Deisseroth, The development and application of optogenetics. *Annu. Rev. Neurosci.* **34**, 389–412 (2011).

3. Y. Nihongaki, F. Kawano, T. Nakajima, M. Sato, Photoactivatable CRISPR-Cas9 for optogenetic genome editing. *Nat. Biotechnol.* **33**, 755–760 (2015).

4. Z. Huang, Y. Wu, M. E. Allen, Y. Pan, P. Kyriakakis, S. Lu, Y.-J. Chang, X. Wang, S. Chien, Y. Wang, Engineering light-controllable CAR T cells for cancer immunotherapy. *Sci. Adv.* **6**, eaay9209 (2020).

5. S. H. Yun, S. J. J. Kwok, Light in diagnosis, therapy and surgery. *Nat. Biomed. Eng.* **1**, 0008 (2017).

6. Y. Yang, M. Wu, A. J. Wegener, A. Vázquez-Guardado, A. I. Efimov, F. Lie, T. Wang, Y. Ma, A. Banks, Z. Li, Z. Xie, Y. Huang, C. H. Good, Y. Kozorovitskiy, J. A. Rogers, Preparation and use of wireless reprogrammable multilateral optogenetic devices for behavioral neuroscience. *Nat. Protoc.* **17**, 1073–1096 (2022).

7. A. D. Mickle, S. M. Won, K. N. Noh, J. Yoon, K. W. Meacham, Y. Xue, L. A. McIlvried, B. A. Copits, V. K. Samineni, K. E. Crawford, K. Do Hoon, P. Srivastava, B. H. Kim, S. Min, Y. Shiuan, Y. Yun, M. A. Payne, J. Zhang, H. Jang, Y. Li, H. Henry Lai, Y. Huang, S.-I. Park, R. W. Gereau, J. A. Rogers, A wireless closed-loop system for optogenetic peripheral neuromodulation. *Nature* **565**, 361–365 (2019).

8. T. Maldiney, A. Bessière, J. Seguin, E. Teston, S. K. Sharma, B. Viana, A. J. J. Bos, P. Dorenbos, M. Bessodes, D. Gourier, D. Scherman, C. Richard, The in vivo activation of persistent nanophosphors for optical imaging of vascularization, tumours and grafted cells. *Nat. Mater.* **13**, 418–426 (2014).

9. Q. Miao, C. Xie, X. Zhen, Y. Lyu, H. Duan, X. Liu, J. V. Jokerst, K. Pu, Molecular afterglow imaging with bright, biodegradable polymer nanoparticles. *Nat. Biotechnol.* **35**, 1102–1110 (2017).

10. Z. Li, Y. Zhao, K. Huang, L. Huang, Y. Zhang, H. Yang, G. Han, Enhancing rechargeable persistent luminescence via organic dye sensitization. *Angew. Chem. Int. Ed. Engl.* **60**, 15886–15890 (2021).

11. Y. Jiang, J. Huang, X. Zhen, Z. Zeng, J. Li, C. Xie, Q. Miao, J. Chen, P. Chen, K. Pu, A generic approach towards afterglow luminescent nanoparticles for ultrasensitive in vivo imaging. *Nat. Commun.* **10**, 2064 (2019).

12. R. N. Day, M. W. Davidson, The fluorescent protein palette: Tools for cellular imaging. *Chem. Soc. Rev.* **38**, 2887–2921 (2009).

13. X. X. Zhou, X. Zou, H. K. Chung, Y. Gao, Y. Liu, L. S. Qi, M. Z. Lin, A single-chain photoswitchable CRISPR-Cas9 architecture for light-inducible gene editing and transcription. *ACS Chem. Biol.* **13**, 443–448 (2018).

14. X. Su, X. Kong, K. Sun, Q. Liu, Y. Pei, D. Hu, M. Xu, W. Feng, F. Li, Enhanced blue afterglow through molecular fusion for bio-applications. *Angew. Chem. Int. Ed. Engl.*, e202201630 (2022).

15. L. Ma, X. Zou, M. Hossu, W. Chen, Synthesis of ZnS:Ag,Co water-soluble blue afterglow nanoparticles and application in photodynamic activation. *Nanotechnology* **27**, 315602 (2016).

16. P. Pei, Y. Chen, C. Sun, Y. Fan, Y. Yang, X. Liu, L. Lu, M. Zhao, H. Zhang, D. Zhao, X. Liu, F. Zhang, X-ray-activated persistent luminescence nanomaterials for NIR-II imaging. *Nat. Nanotechnol.* **16**, 1011–1018 (2021).

17. Z. Li, Y. Zhang, X. Wu, L. Huang, D. Li, W. Fan, G. Han, Direct aqueous-phase synthesis of sub-10 nm "Luminous Pearls" with enhanced in vivo renewable near-infrared persistent luminescence. *J. Am. Chem. Soc.* **137**, 5304–5307 (2015).

18. Q. L. M. De Chermont, C. Chanéac, J. Seguin, F. Pellé, S. Maîtrejean, J.-P. Jolivet, D. Gourier, M. Bessodes, D. Scherman, Nanoprobes with near-infrared persistent luminescence for in vivo imaging. *Proc. Natl. Acad. Sci. U.S.A.* **104**, 9266–9271 (2007).

19. F. Liu, W. Yan, Y.-J. Chuang, Z. Zhen, J. Xie, Z. Pan, Photostimulated near-infrared persistent luminescence as a new optical read-out from Cr^{3+}-doped $LiGa_5O_8$. *Sci. Rep.* **3**, 1554 (2013).

20. Y. Li, M. Gecevicius, J. Qiu, Long persistent phosphors—From fundamentals to applications. *Chem. Soc. Rev.* **45**, 2090–2136 (2016).

21. P. F. Smet, K. Van den Eeckhout, O. Q. De Clercq, D. Poelman, Persistent phosphors, in *Handbook on the Physics and Chemistry of Rare Earths* (Elsevier, 2015), vol. 48, pp. 1–108.

22. C. Suryanarayana, Mechanical alloying and milling. *Prog. Mater Sci.* **46**, 1–184 (2001).

23. D. Tu, C.-N. Xu, Y. Fujio, S. Kamimura, Y. Sakata, N. Ueno, Phosphorescence quenching by mechanical stimulus in CaZnOS:Cu. *Appl. Phys. Lett.* **105**, 011908 (2014).

24. X. Qin, X. Liu, W. Huang, M. Bettinelli, X. Liu, Lanthanide-activated phosphors based on 4f-5d optical transitions: Theoretical and experimental aspects. *Chem. Rev.* **117**, 4488–4527 (2017).

25. R. Tang, L. Wang, C. A. Orme, T. Bonstein, P. J. Bush, G. H. Nancollas, Dissolution at the nanoscale: Self-preservation of biominerals. *Angew. Chem. Int. Ed. Engl.* **116**, 2751–2755 (2004).

26. D. Athanasiadou, W. Jiang, D. Goldbaum, A. Saleem, K. Basu, M. S. Pacella, C. F. Böhm, R. R. Chromik, M. T. Hincke, A. B. Rodríguez-Navarro, H. Vali, S. E. Wolf, J. J. Gray, K. H. Bui, M. D. McKee, Nanostructure, osteopontin, and mechanical properties of calcitic avian eggshell. *Sci. Adv.* **4**, eaar3219 (2018).

27. R. Tang, G. H. Nancollas, C. A. Orme, Mechanism of dissolution of sparingly soluble electrolytes. *J. Am. Chem. Soc.* **123**, 5437–5443 (2001).

28. P. M. Dove, N. Han, J. J. De Yoreo, Mechanisms of classical crystal growth theory explain quartz and silicate dissolution behavior. *Proc. Natl. Acad. Sci. U.S.A.* **102**, 15357–15362 (2005).

29. L. Ratke, P. W. Voorhees, *Growth and Coarsening: Ostwald Ripening in Material Processing* (Springer Science & Business Media, 2013).

30. W. Wu, G. H. Nancollas, Determination of interfacial tension from crystallization and dissolution data: A comparison with other methods. *Adv. Colloid Interface Sci.* **79**, 229–279 (1999).

31. E. S. Boyden, F. Zhang, E. Bamberg, G. Nagel, K. Deisseroth, Millisecond-timescale, genetically targeted optical control of neural activity. *Nat. Neurosci.* **8**, 1263–1268 (2005).

32. I. P. Sahu, D. P. Bisen, N. Brahme, R. Sharma, Luminescence properties of Eu2, Dy3 -doped Sr2MgSi2O7, and Ca2MgSi2O7 phosphors by solid-state reaction method. *Res. Chem. Intermed.* **41** 6649–6664 (2015).

33. Q. Zhou, M. Xu, W. Feng, F. Li, Quantum yield measurements of photochemical reaction-based afterglow luminescence materials. *J. Phys. Chem. Lett.* **12**, 9455–9462 (2021).

34. Y. Gao, R. Li, W. Zheng, X. Shang, J. Wei, M. Zhang, J. Xu, W. You, Z. Chen, X. Chen, Broadband NIR photostimulated luminescence nanoprobes based on CaS:Eu^{2+}, Sm^{3+} nanocrystals. *Chem. Sci.* **10**, 5452–5460 (2019).

35. Y. Lin, Z. Tang, Z. Zhang, C. W. Nan, Anomalous luminescence in $Sr_4Al_{14}O_{25}$:Eu, Dy phosphors. *Appl. Phys. Lett.* **81**, 996–998 (2002).

36. H. Li, S. Yin, Y. Wang, T. Sekino, S. W. Lee, T. Sato, Green phosphorescence-assisted degradation of rhodamine Bdyes by Ag3PO4. *J. Mater. Chem. A Mater. Energy Sustain.* **1**, 1123–1126 (2013).

37. C. Zhang, M. Zhang, W. Zheng, J. Wei, S. Wang, P. Huang, X. Cheng, T. Dai, Z. Chen, X. Chen, A new class of luminescent nanoprobes based on main-group Sb^{3+} emitters. *Nano Res.* **15**, 179–185 (2022).

38. J. Wang, Y. Zhu, C. A. Grimes, Q. Cai, Multicolor lanthanide-doped CaS and SrS near-infrared stimulated luminescent nanoparticles with bright emission: Application in broad-spectrum lighting, information coding, and bio-imaging. *Nanoscale* **11**, 12497–12501 (2019).

39. X. Qiu, X. Zhu, M. Xu, W. Yuan, W. Feng, F. Li, Hybrid nanoclusters for near-infrared to near-infrared upconverted persistent luminescence bioimaging. *ACS Appl. Mater. Interfaces* **9**, 32583–32590 (2017).

40. W. Yang, R. Yuste, In vivo imaging of neural activity. *Nat. Methods* **14**, 349–359 (2017).

41. J. K. Adams, V. Boominathan, S. Gao, A. V. Rodriguez, D. Yan, C. Kemere, A. Veeraraghavan, J. T. Robinson, *In vivo* fluorescence imaging with a flat, lensless microscope. bioRxiv 2020.06.04.135236 [**Preprint**]. 25 June 2020. https://doi.org/10.1101/2020.06.04.135236.

42. G. Feng, R. H. Mellor, M. Bernstein, C. Keller-Peck, Q. T. Nguyen, M. Wallace, J. M. Nerbonne, J. W. Lichtman, J. R. Sanes, Imaging neuronal subsets in transgenic mice expressing multiple spectral variants of GFP. *Neuron* **28**, 41–51 (2000).

43. K. W. Dunn, M. M. Kamocka, J. H. McDonald, A practical guide to evaluating colocalization in biological microscopy. *Am. J. Physiol. Cell Physiol.* **300**, C723–C742 (2011).

44. X. Wu, X. Zhu, P. Chong, J. Liu, L. N. Andre, K. S. Ong, K. Brinson Jr., A. I. Mahdi, J. Li, L. E. Fenno, H. Wang, G. Hong, Sono-optogenetics facilitated by a circulation-delivered rechargeable light source for minimally invasive optogenetics. *Proc. Natl. Acad. Sci. U.S.A.* **116**, 26332–26342 (2019).

45. J. Szanics, T. Okubo, M. Kakihana, Preparation of $LiTaO_3$ powders at reduced temperatures by a polymerized complex method. *J. Alloys Compd.* **281**, 206–210 (1998).

46. L. H. Wang, D. R. Yuan, X. L. Duan, X. Q. Wang, F. P. Yu, Synthesis and characterization of fine lithium niobate powders by sol- gel method. *Cryst. Res. Technol.* **42**, 321–324 (2007).

47. A. Singer, S. Dutta, E. Lewis, Z. Chen, J. C. Chen, N. Verma, B. Avants, A. K. Feldman, J. O'Malley, M. Beierlein, C. Kemere, J. T. Robinson, Magnetoelectric materials for miniature, wireless neural stimulation at therapeutic frequencies. *Neuron* **107**, 631–643.e5 (2020).

48. X. Ou, X. Qin, B. Huang, J. Zan, Q. Wu, Z. Hong, L. Xie, H. Bian, Z. Yi, X. Chen, Y. Wu, X. Song, J. Li, Q. Chen, H. Yang, X. Liu, High-resolution x-ray luminescence extension imaging. *Nature* **590**, 410–415 (2021).

49. N. Kohler, G. E. Fryxell, M. Zhang, A bifunctional poly(ethylene glycol) silane immobilized on metallic oxide-based nanoparticles for conjugation with cell targeting agents. *J. Am. Chem. Soc.* **126**, 7206–7211 (2004).

50. Y. Zhong, Z. Ma, F. Wang, X. Wang, Y. Yang, Y. Liu, X. Zhao, J. Li, H. Du, M. Zhang, Q. Cui, S. Zhu, Q. Sun, H. Wan, Y. Tian, Q. Liu, W. Wang, K. C. Garcia, H. Dai, In vivo molecular imaging for immunotherapy using ultra-bright near-infrared-IIb rare-earth nanoparticles. *Nat. Biotechnol.* **37**, 1322–1331 (2019).

51. J. Cohen, *Statistical Power Analysis for the Behavioral Sciences* (Academic Press, 2013).

52. T. Liu, L. Li, X. Teng, X. Huang, H. Liu, D. Chen, J. Ren, J. He, F. Tang, Single and repeated dose toxicity of mesoporous hollow silica nanoparticles in intravenously exposed mice. *Biomaterials* **32**, 1657–1668 (2011).

53. F. J. Martin, K. Melnik, T. West, J. Shapiro, M. Cohen, A. A. Boiarski, M. Ferrari, Acute toxicity of intravenously administered microfabricated silicon dioxide drug delivery particles in mice: Preliminary findings. *Drugs R D* **6**, 71–81 (2005).

54. X. Wu, Y. Jiang, N. J. Rommelfanger, F. Yang, Q. Zhou, R. Yin, J. Liu, S. Cai, W. Ren, A. Shin, K. S. Ong, K. Pu, G. Hong, Tether-free photothermal deep-brain stimulation in freely behaving mice via wide-field illumination in the near-infrared-II window. *Nat. Biomed. Eng.* **6**, 754–770 (2022),.

55. O. Yizhar, L. E. Fenno, M. Prigge, F. Schneider, T. J. Davidson, D. J. O'Shea, V. S. Sohal, I. Goshen, J. Finkelstein, J. T. Paz, K. Stehfest, R. Fudim, C. Ramakrishnan, J. R. Huguenard, P. Hegemann, K. Deisseroth, Neocortical excitation/inhibition balance in information processing and social dysfunction. *Nature* **477**, 171–178 (2011).

56. A. Berndt, O. Yizhar, L. A. Gunaydin, P. Hegemann, K. Deisseroth, Bi-stable neural state switches. *Nat. Neurosci.* **12**, 229–234 (2009).

57. X. Gong, D. Mendoza-Halliday, J. T. Ting, T. Kaiser, X. Sun, A. M. Bastos, R. D. Wimmer, B. Guo, Q. Chen, Y. Zhou, M. Pruner, C. W.-H. Wu, D. Park, K. Deisseroth, B. Barak, E. S. Boyden, E. K. Miller, M. M. Halassa, Z. Fu, G. Bi, R. Desimone, G. Feng, An ultra-sensitive step-function opsin for minimally invasive optogenetic stimulation in mice and macaques. *Neuron.* **107**, 38–51.e8 (2020).

58. B. A. Hartl, H. Hirschberg, L. Marcu, S. R. Cherry, Characterizing low fluence thresholds for in vitro photodynamic therapy. *Biomed. Opt. Express.* **6**, 770–779 (2015).

59. E. H. Kim, S. Park, Y. K. Kim, M. Moon, J. Park, K. J. Lee, S. Lee, Y.-P. Kim, Self-luminescent photodynamic therapy using breast cancer targeted proteins. *Sci Adv.* **6**, eaba3009 (2020).

60. J. Schubert, A. Lindenbaum, Stability of alkaline earth—Organic acid complexes measured by ion exchange. *J. Am. Chem. Soc.* **74**, 3529–3532 (1952).

61. J. L. Meyer, Formation constants for interaction of citrate with calcium and magnesium lons. *Anal. Biochem.* **62**, 295–300 (1974).

62. P. C. Bennett, M. E. Melcer, D. I. Siegel, J. P. Hassett, The dissolution of quartz in dilute aqueous solutions of organic acids at 25°C. *Geochim. Cosmochim. Acta* **52**, 1521–1530 (1988).

63. A. Heller, A. Barkleit, H. Foerstendorf, S. Tsushima, K. Heim, G. Bernhard, Curium(III) citrate speciation in biological systems: A europium(iii) assisted spectroscopic and quantum chemical study. *Dalton Trans.* **41**, 13969–13983 (2012).

64. D. T. Sawyer, P. J. Paulsen, Properties and infrared spectra of ethylenediaminetetraacetic acid complexes. II. Chelates of divalent ions. *J. Am. Chem. Soc.* **81**, 816–820 (1959).

65. A. Gácsi, B. Kutus, Á. Buckó, Z. Csendes, G. Peintler, I. Pálinkó, P. Sipos, Some aspects of the aqueous solution chemistry of the $Na^{+}/Ca^{2+}/OH^{-}/Cit^{3-}$ system: The structure of a new calcium citrate complex forming under hyperalkaline conditions. *J. Mol. Struct.* **1118,** 110–116 (2016).

66. A. Bodor, I. Bányai, L. Zékány, I. Tóth, Slow dynamics of aluminium-citrate complexes studied by 1H- and 13C-NMR spectroscopy. *Coord. Chem. Rev.* **228**, 163–173 (2002).

67. F. R. Bacon, F. C. Raggon, Promotion of attack on glass and silica by citrate and other anions in neutral solution. *J. Am. Ceram. Soc.* **42**, 199–205 (1959).

68. L. Zamirri, A. M. Escatllar, J. M. Guiu, P. Ugliengo, S. T. Bromley, What can infrared spectra tell us about the crystallinity of nanosized interstellar silicate dust grains? *ACS Earth Space Chem.* **3**, 2323–2338 (2019).

69. A. I. Bortun, L. N. Bortun, A. Clearfield, Hydrothermal synthesis of sodium zirconium silicates and characterization of their properties. *Chem. Mater.* **9**, 1854–1864 (1997).

70. Z. Li, N. Yu, J. Zhou, Y. Li, Y. Zhang, L. Huang, K. Huang, Y. Zhao, S. Kelmar, J. Yang, G. Han, Coloring afterglow nanoparticles for high-contrast time-gating-free multiplex luminescence imaging. *Adv. Mater.* **32**, 2003881 (2020).

71. Y.-J. Li, C.-X. Yang, X.-P. Yan, Biomimetic persistent luminescent nanoplatform for autofluorescence-free metastasis tracking and chemophotodynamic therapy. *Anal. Chem.* **90**, 4188–4195 (2018).

72. Y.-F. Gao, R. Zou, G.-F. Chen, B.-M. Liu, Y. Zhang, J. Jiao, K.-L. Wong, J. Wang, Large-pore mesoporous-silica-assisted synthesis of high-performance $ZnGa_2O_4:Cr^{3+}/Sn^{4+}$@MSNs multifunctional nanoplatform with optimized optical probe mass ratio and superior residual pore volume for improved bioimaging and drug delivery. *Chem. Eng. J.* **420**, 130021 (2021).

73. Y. Wang, C.-X. Yang, X.-P. Yan, Hydrothermal and biomineralization synthesis of a dual-modal nanoprobe for targeted near-infrared persistent luminescence and magnetic resonance imaging. *Nanoscale* **9**, 9049–9055 (2017).

74. A. Abdukayum, J.-T. Chen, Q. Zhao, X.-P. Yan, Functional near infrared-emitting Cr^{3+}/Pr^{3+} co-doped zinc gallogermanate persistent luminescent nanoparticles with superlong afterglow for *in vivo* targeted bioimaging. *J. Am. Chem. Soc.* **135**, 14125–14133 (2013).

75. Y. Feng, R. Liu, L. Zhang, Z. Li, Y. Su, Y. Lv, Raspberry-like mesoporous $Zn_{1.07}Ga_{2.34}Si_{0.98}O_{6.56}:Cr_{0.01}$ nanocarriers for enhanced near-infrared afterglow imaging and combined cancer chemotherapy. *ACS Appl. Mater. Interfaces* **11**, 44978–44988 (2019).

76. Y.-J. Li, X.-P. Yan, Synthesis of functionalized triple-doped zinc gallogermanate nanoparticles with superlong near-infrared persistent luminescence for long-term orally administrated bioimaging. *Nanoscale* **8,** 14965–14970 (2016).

77. J. Shi, X. Sun, S. Zheng, J. Li, X. Fu, H. Zhang, A new near-infrared persistent luminescence nanoparticle as a multifunctional nanoplatform for multimodal imaging and cancer therapy. *Biomaterials* **152**, 15–23 (2018).

78. Y. Lv, D. Ding, Y. Zhuang, Y. Feng, J. Shi, H. Zhang, T.-L. Zhou, H. Chen, R.-J. Xie, Chromium-doped zinc gallogermanate@zeolitic imidazolate framework-8: A multifunctional nanoplatform for rechargeable in vivo persistent luminescence imaging and ph-responsive drug release. *ACS Appl. Mater. Interfaces* **11**, 1907–1916 (2019).

79. M. Pellerin, E. Glais, T. Lecuyer, J. Xu, J. Seguin, S. Tanabe, C. Chanéac, B. Viana, C. Richard, $LaAlO_3:Cr^{3+}$, Sm^{3+}: Nano-perovskite with persistent luminescence for *in vivo* optical imaging. *J. Lumin.* **202**, 83–88 (2018).

80. T. Ai, W. Shang, H. Yan, C. Zeng, K. Wang, Y. Gao, T. Guan, C. Fang, J. Tian, Near infrared-emitting persistent luminescent nanoparticles for Hepatocellular Carcinoma imaging and luminescence-guided surgery. *Biomaterials* **167**, 216–225 (2018).

81. T. Maldiney, A. Lecointre, B. Viana, A. Bessière, M. Bessodes, D. Gourier, C. Richard, D. Scherman, Controlling electron trap depth to enhance optical properties of persistent luminescence nanoparticles for in vivo imaging. *J. Am. Chem. Soc.* **133**, 11810–11815 (2011).

82. Z. Xue, X. Li, Y. Li, M. Jiang, H. Liu, S. Zeng, J. Hao, X-ray-activated near-infrared persistent luminescent probe for deep-tissue and renewable in vivo bioimaging. *ACS Appl. Mater. Interfaces* **9**, 22132–22142 (2017).

83. B. Zheng, Y. Bai, H. Chen, H. Pan, W. Ji, X. Gong, X. Wu, H. Wang, J. Chang, Near-infrared light-excited upconverting persistent nanophosphors in vivo for imaging-guided cell therapy. *ACS Appl. Mater. Interfaces* **10**, 19514–19522 (2018).

84. X. Chen, Y. Li, K. Huang, L. Huang, X. Tian, H. Dong, R. Kang, Y. Hu, J. Nie, J. Qiu, G. Han, Trap energy upconversion-like near-infrared to near-infrared light rejuvenateable persistent luminescence. *Adv. Mater.* **33**, 2008722 (2021).

85. C. Xie, X. Zhen, Q. Miao, Y. Lyu, K. Pu, Self-assembled semiconducting polymer nanoparticles for ultrasensitive near-infrared afterglow imaging of metastatic tumors. *Adv. Mater.* **30**, 1801331 (2018).

86. X. Zhen, Y. Tao, Z. An, P. Chen, C. Xu, R. Chen, W. Huang, K. Pu, Ultralong phosphorescence of water-soluble organic nanoparticles for in vivo afterglow imaging. *Adv. Mater.* **29**, 1606665 (2017).

87. F. Zhang, M. Xu, X. Su, W. Yuan, W. Feng, Q. Su, F. Li, Afterglow implant for arterial embolization and intraoperative imaging. *Chemistry* **28**, e202103795 (2022).

88. X. Wang, W. Yuan, M. Xu, X. Su, F. Li, Visualization of acute inflammation through a macrophage-camouflaged afterglow nanocomplex. *ACS Appl. Mater. Interfaces* **14**, 259–267 (2022).

89. X. Ni, X. Zhang, X. Duan, H.-L. Zheng, X.-S. Xue, D. Ding, Near-infrared afterglow luminescent aggregation-induced emission dots with ultrahigh tumor-to-liver signal ratio for promoted image-guided cancer surgery. *Nano Lett.* **19**, 318–330 (2019).
